# Autophagy of mucin granules contributes to resolution of airway mucous metaplasia

**DOI:** 10.1038/s41598-021-91932-7

**Published:** 2021-06-22

**Authors:** J. M. Sweeter, K. Kudrna, K. Hunt, P. Thomes, B. F. Dickey, S. L. Brody, J. D. Dickinson

**Affiliations:** 1 Pulmonary, Critical Care and Sleep Medicine Division, Department of Internal Medicine, University of Nebraska Medical Center, Omaha, NE USA; 2 Department of Pulmonary Medicine, MD Anderson Cancer Center, Houston, TX USA; 3 Department of Medicine, Washington University School of Medicine, Saint Louis, MO USA

**Keywords:** Autophagy, Chronic obstructive pulmonary disease, Cystic fibrosis, Mechanisms of disease, Innate immunity, Cell death and immune response

## Abstract

Exacerbations of muco-obstructive airway diseases such as COPD and asthma are associated with epithelial changes termed mucous metaplasia (MM). Many molecular pathways triggering MM have been identified; however, the factors that regulate resolution are less well understood. We hypothesized that the autophagy pathway is required for resolution of MM by eliminating excess non-secreted intracellular mucin granules. We found increased intracellular levels of mucins Muc5ac and Muc5b in mice deficient in autophagy regulatory protein, Atg16L1, and that this difference was not due to defects in the known baseline or stimulated mucin secretion pathways. Instead, we found that, in mucous secretory cells, Lc3/Lamp1 vesicles colocalized with mucin granules particularly adjacent to the nucleus, suggesting that some granules were being eliminated in the autophagy pathway rather than secreted. Using a mouse model of MM resolution, we found increased lysosomal proteolytic activity that peaked in the days after mucin production began to decline. In purified lysosomal fractions, Atg16L1-deficient mice had reduced proteolytic degradation of Lc3 and Sqstm1 and persistent accumulation of mucin granules associated with impaired resolution of mucous metaplasia. In normal and COPD derived human airway epithelial cells (AECs), activation of autophagy by mTOR inhibition led to a reduction of intracellular mucin granules in AECs. Our findings indicate that during peak and resolution phases of MM, autophagy activity rather than secretion is required for elimination of some remaining mucin granules. Manipulation of autophagy activation offers a therapeutic target to speed resolution of MM in airway disease exacerbations.

## Introduction

Mammalian cells utilize two primary degradation systems to recycle proteins: the proteasome and the autophagosome-lysosome pathways. While there is some overlap, these pathways are largely independently regulated and serve different functions^1^. Protein degradation systems are necessary for the cell to balance metabolic demands by recycling proteins to amino acids for new synthesis. The autophagosome-lysosome pathway is highly conserved across species^2,3^. It can be utilized in bulk protein breakdown (macro-autophagy or referred to as autophagy) in response to nutrient demands for new amino acids. Cells can also utilize chaperone-mediated autophagy that utilizes a specific amino acid motif targeted by cytoplasmic chaperones that bypass the autophagosome and directly insert proteins into the lysosome for degradation. Cells can also utilize selective autophagy by targeting proteins, protein aggregates, and organelles such as mitochondria^4,5^ or cilia components^6^ to the autophagosome for degradation in the lysosome. We^7,8^, and others^9–13^, have observed that autophagy protein markers are increased in models of human airway disease, and in asthma and chronic obstructive pulmonary disease (COPD) airways. This led us to infer that autophagy played a key role in the response of the epithelium to airway inflammation.

Mucociliary clearance is a vital feature of innate immunity^14,15^. There are two primary secretory mucins in murine and human airways, Muc5b/MUC5B and Muc5ac/MUC5AC, that impart the biophysical properties of the mucus gel layer^16,17^. MUC5B is the homeostatic mucin tonically produced and secreted throughout the airways to facilitate mucocilary clearance^18^. In contrast, MUC5AC production is highly inducible in response to certain types of airway inflammation^19,20^. Mucins are large proteins, heavily glycosylated in the Golgi, and then packaged in granules for apical transport to the plasma membrane where they await secretion to the airway lumen. Mucin granules can undergo baseline or stimulated secretion^21–26^, and rely on distinct exocytic molecular machines for the two different release rates^27^.

In muco-obstructive airway diseases such as asthma, COPD, cystic fibrosis (CF), and non-CF bronchiectasis, increased MUC5AC production in response to inflammatory stimuli leads to extensive changes of the airway epithelium, termed mucous metaplasia (MM) marked by enlargement of secretory cells with increased intracellular staining of MUC5AC granules. Airway inflammation also provides stimuli, such as ATP, for enhanced mucin secretion into the airway lumen leading to airway occlusion and loss of lung function^19,28^. The factors that regulate the development of mucous metaplasia in muco-obstructive airway diseases have been extensively characterized in mouse and human airway models. Mouse secretory epithelial cells, marked by intracellular Secretoglobin 1a1 and Muc5b, along with proteins involved in the regulated exocytic machinery, transition from a club cell appearance to a goblet cell morphology with cytoplasm packed with Muc5ac granules^21^. A number of inflammatory and allergen factors have been identified that initiate mucous metaplasia of the airway epithelium. These include Type 2 inflammatory cytokines such as IL-13^29–31^, growth and development factors such as NOTCH^32–34^ and EGF^35^, and respiratory viruses such as rhinovirus^36^. The transcriptional activity of Spdef^37,38^ plays a central role downstream of many of the signals to induce mucous metaplasia. What has not been fully studied is how the airway secretory cell normalizes as airway inflammation abates. In rat models challenged with LPS in the airway, the anti-apoptotic protein, Bcl2, is increased to support cellular hyperplasia^39,40^ with an approximately 30% increase in secretory cells number. In contrast, in the mouse airway model, there is only a negligible number of proliferating cells or expansion of secretory cell number with OVA mediated by mucous metaplasia^41^.

We chose to address how the resolution of excess mucin granules occurs in secretory cells, which is a distinct phenomenon from resolution of the epithelial hyperplasia observed in some airway models. We considered that during resolution of mucous metaplasia, as secretory cell size diminishes, mucin granules may continue to be secreted, or alternatively, degraded. Here, we show that mice globally deficient in the autophagy regulatory protein, Atg16L1, have increased Lc3 II and Sqstm1 in the lysosome compartment and higher levels of mucin granules in both intracellular and lysosome enrich preparations during resolution of mucous metaplasia. We also found that autolysosomes (Lc3 and Lamp1 labeled vesicles) closely approximate and engulf mucin granules. Finally, we demonstrate that induction of autophagy through mTOR inhibition leads to reduced intracellular MUC5AC in human AECs. Together, this indicates that the autophagy-lysosome pathway is required to resolve airway mucous metaplasia by removing excess mucin granules.

## Results

### Atg16L1^HM^ mice have increased airway Muc5ac and Muc5b levels during mucous metaplasia

**Figure 1:**
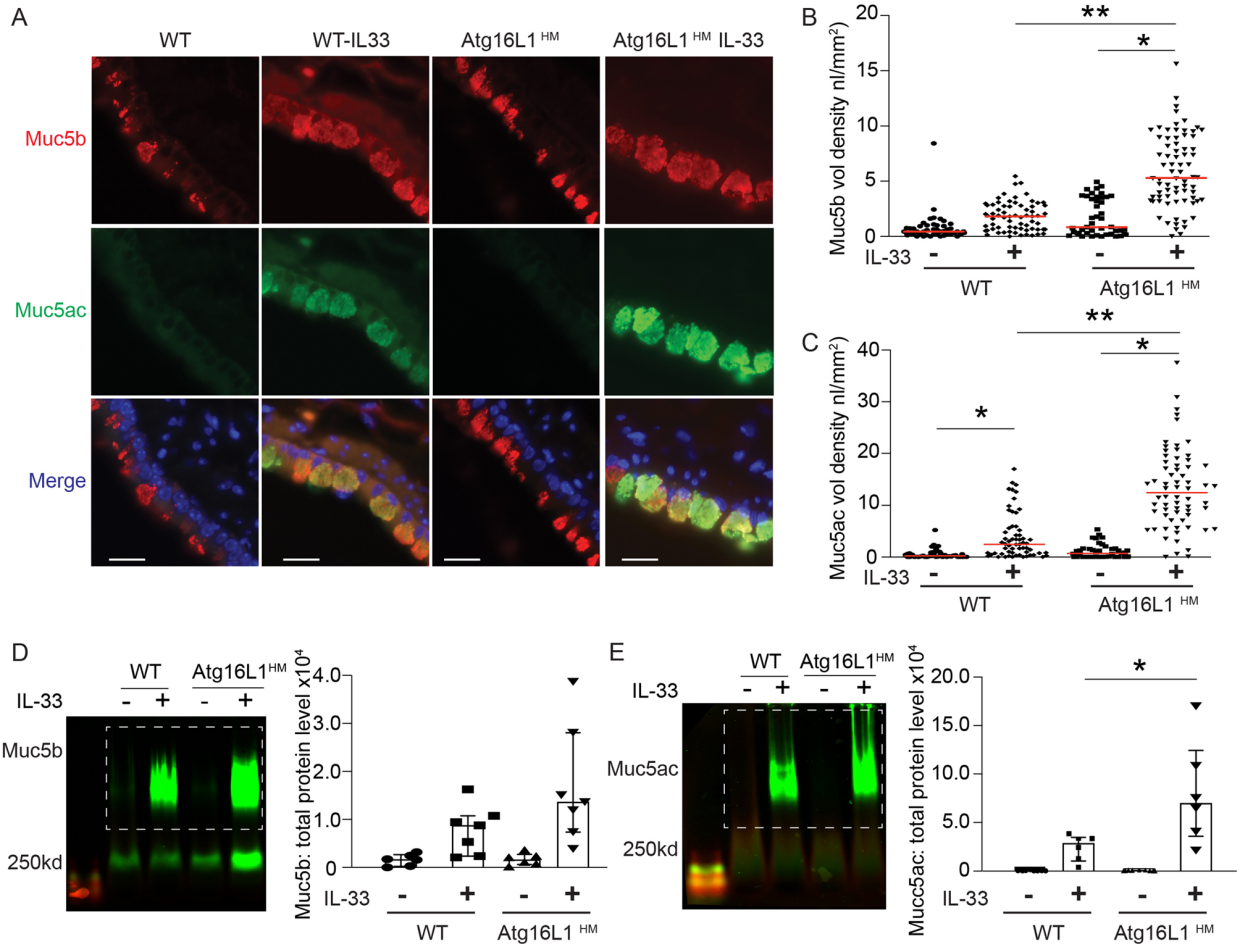
Atg16L1 deficient mice have increased mucin levels with IL-33-mediated mucous metaplasia. (**A**) Representative images of immunostaining for mouse anti-Muc5b (red) and Muc5ac by lectin UEA-1 (green) are shown. Nuclei are countered stained with DAPI in blue. Scale bar equals 20 microns. Quantification of Muc5b (**B**) and lectin UEA-1/Muc5ac (**C**) airways volume density (nl/mm^2^ (n = 6 mice per group). (**D**, **E**) Representative immunoblots of lung homogenates of Muc5b and Muc5ac by lectin UEA-1 from Atg16L1^HM^ or WT mice with corresponding quantification normalized to total protein level (n = 7 mice per group). Dashed box denotes area of mucin quantification. Graphs show scatter plots with median bar for mucin immunostaining and scatter plots with median and interquartile range distribution bars for mucin blots. Significant difference by ANOVA with Tukey’s post-hoc comparisons are noted by * for IL-33 treatment difference or ** for mouse genotype differences for immunostaining and parts (**B**) and (**C**). Significant difference by Mann–Whitney test for Muc5ac/UEA-1 western blots between OVA challenged WT and Atg16L1^HM^ mice in Part (**E**).

We previously showed that there were increased intracellular MUC5AC-positive granules when autophagy protein, ATG5, was depleted by shRNA in human AECs cultured with IL-13^7^. In addition, we found that LC3 levels were increased as measured by LC3 II accumulation during flux assay in human AECs challenged with Type 2 cytokines IL-13 and IL-4^7,8^. ATG5-12 and ATG16L1 form a complex that facilitates autophagosome maturation. We therefore utilized mice globally deficient in Atg16L1 (Atg16L1^HM^), which have reduced degradation of the autophagy target Lc3 II in the lungs (Fig. S1A,B) and increased lysosomal accumulation of Lc3 II and Sqstm1 (Fig. S1C) in naïve non-inflamed mice, consistent with previous findings in the mouse ileum^42^. To explore the effect of reduced autophagy-mediated degradation during states of significant mucin production and intracellular accumulation, we stimulated wildtype (WT) or Atg16L1^HM^ mice with 3 intra-nasal IL-33 challenges over 7 days. IL-33 was used to activate immune cells to release endogenous IL-13 which leads to robust airway mucous metaplasia as we^7^, and others^43,44^, have previously shown. To detect airway mucins, we relied on a monoclonal antibody for Muc5b detection^25,27^ and lectin UEA-1 for Muc5ac detection^37,46^. Atg16L16^HM^ mice had increased intracellular Muc5ac granules and, to a lesser extent, intracellular Muc5b granules by immunostaining (Fig. 1A–C) and increased overall lung homogenate Muc5ac levels by immunoblotting compared to WT mice (Fig. 1D,E). We found a similar result using rabbit polyclonal antibody against Muc5ac by immunostaining (Fig. S2A,B). Therefore, lectin UEA-1 staining was used as a marker for Muc5ac in mouse tissue for subsequent staining and immunoblotting. These findings indicate the mice globally deficient in autophagy protein, Atg16L1, have increased accumulation of airway mucins.

### Atg16L1 deficient mice have a normal secretory response

**Figure 2:**
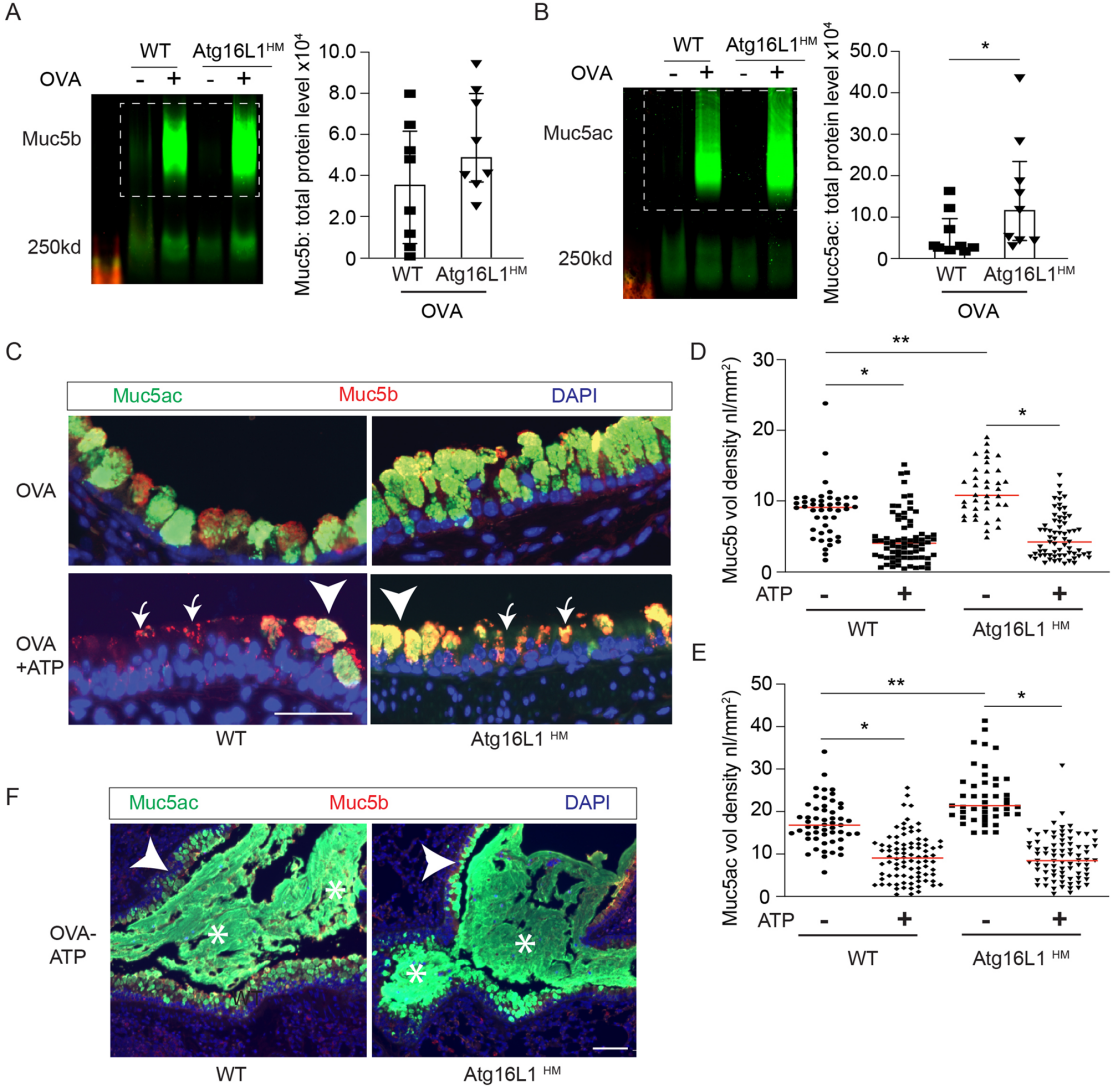
Atg16L1 deficient mice have increased intracellular mucins and normal stimulated mucin secretion. (**A**, **B**) Representative immunoblots of lung homogenates for Muc5b and Muc5ac by lectin UEA-1 from OVA challenged WT or Atg16L1^HM^ mice with corresponding quantification normalized to total protein level (n = 8 WT mice and 9 Atg16L1^HM^ mice). Dashed box denotes area of mucin quantification. (**C**) Representative images of mucin immunostaining of airways by mouse anti-Muc5b and Muc5ac by lectin UEA-1 in OVA-challenged Atg16L1 ^HM^ and WT mice ± ATP nebulization with corresponding quantification of mucin levels (nl/mm^2^) for Muc5b (**D**) and UEA-1/Muc5ac (**E**) (n = 6 mice per group). Arrows point to mucous cells that have recently emptied intracellular mucin while arrow heads show non secreted mucous cells. Scale bar = 50 microns. Low power magnification representative images (**F**) with _*_ showing mucous plugging in the airways. Arrow heads show intracellular mucins remaining in the epithelial secretory cells. Scale bar = 100 microns. Graphs show scatter plots with median bar for mucin immunostaining and scatter plots with median and interquartile range distribution bars for mucin blots. Significant difference by ANOVA with Tukey’s post-hoc comparisons with * for ATP treatment difference and ** for Atg16LHM difference for staining in parts (**C**) and (**D,E**) and Mann–Whitney test for mucin western blots in parts (**A**) and (**B**).

To test if the accumulation of airway mucins in Atg16L1^HM^ mice was related to stimulated mucin secretion, we nebulized ATP immediately before euthanasia in OVA sensitized and challenged mice. As was the case with IL-33 challenge, inflammation by OVA led to increased Muc5ac in the lungs of mice deficient of Atg16L1 and a trend toward increased Muc5b levels (Fig. 2A–D) by immunoblotting of total lung homogenates. When we measured only intracellular mucins by immunostaining, we found significant increases for both mucins in Atg16L1 deficient mice. Aerosolized ATP led to the expected rapid decrease of intracellular mucin granules due to secretion in both the WT and Atg16L1^HM^ mice (Fig. 2C–E). Many of the individual secretory cells, after exposure to ATP, lost the feature of tightly organized circular mucin granules. Instead, there was irregular mucin staining and reduced intensity indicating that secretory cells had released many of the preformed granules in response to ATP. In the airways of both WT and Atg16L1^HM^ mice, we found examples of complete occlusion by mucus plugs, primarily consisting of Muc5ac (Fig. 2F). These data indicate that the accumulation of airway mucins in autophagy-deficient mice was not due to a role of Atg16L1 as a component of the stimulated exocytic machinery.

**Figure 3:**
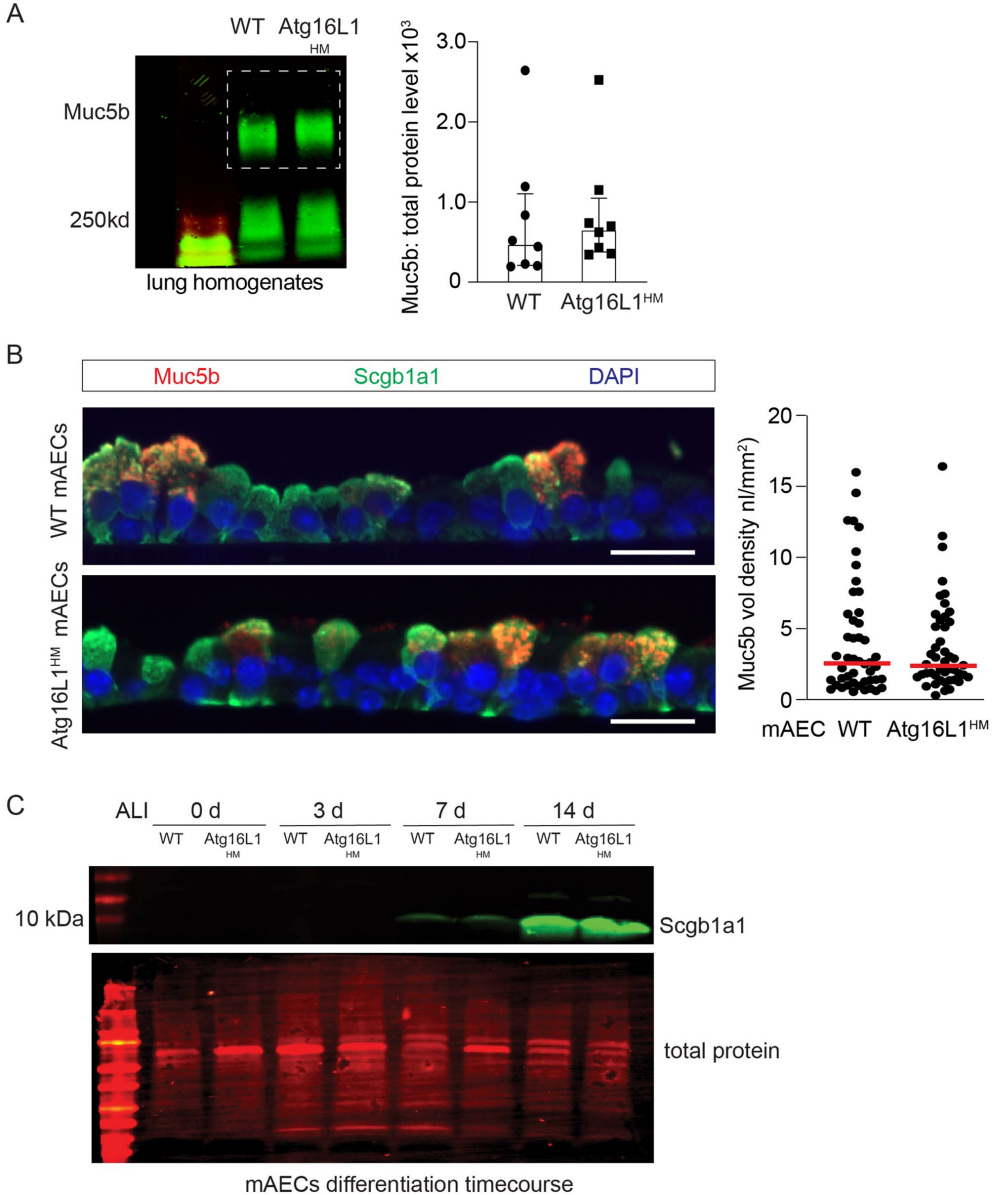
Atg16L1 deficient mice have normal baseline mucin secretion. (**A**) Representative immunoblots with corresponding quantification for Muc5b from lung homogenates from naïve WT and Atg16L1^HM^ mice. Values normalized to total protein levels (n = 7 mice). Dashed box denotes area of mucin quantification. (**B**) Representative Muc5b (red) and Secretoglobin 1a1 (Scgb1a1) (green) immunostaining from WT and Atg16L1^HM^ mouse AECs at ALI day 14 with corresponding Muc5b quantification (n = 4 mTEC inserts from 2 independent preps (scale bar = 25 microns). (**C**) Immunoblot for Secretoglobin 1a1 (Scgb1a1) levels from WT and Atg16L1^HM^ mouse AECs at day 0, 3, 7, and 14 post ALI (blot is representative of two independent mouse AEC preps). Graphs show scatter plots with median bar for mucin immunostaining and scatter plots with median bar and interquartile range for mucin blots.

Secretory cells also rely on baseline mucin secretion with distinct exocytic machinery^22,27,47^, but similarly dependent on tonically released ATP at low levels. As Muc5b is the primary mucin produced and secreted in the naïve mouse airway, we measured Muc5b levels in whole lung homogenates as a marker of baseline mucin secretion in naive WT and Atg16L1^HM^ mice. We found no difference in Muc5b levels in the lung homogenates between WT and Atg16L1^HM^ deficient mice nor in Muc5b intracellular staining in mouse AECs from WT and Atg16L1^HM^ mice differentiated under air liquid interface (ALI) conditions for 14 days (Fig. 3A,B). There was also no significant difference in the levels of secretory cell marker, Scgb1a1, obtained during a time course of ALI differentiation indicating a normal differentiation program in Atg16L1-deficient AECs (Fig. 3C). Together, we deduced that both baseline and stimulated secretion pathways remain intact and secretory cells differentiate normally in Atg16L1-deficient mice. We therefore explored a potential role for autophagy activity in eliminating excess mucin granules from the cytoplasm during resolution.

### Airway mucous cells are highly enriched for lysosomes that congregate around mucin granules

**Figure 4:**
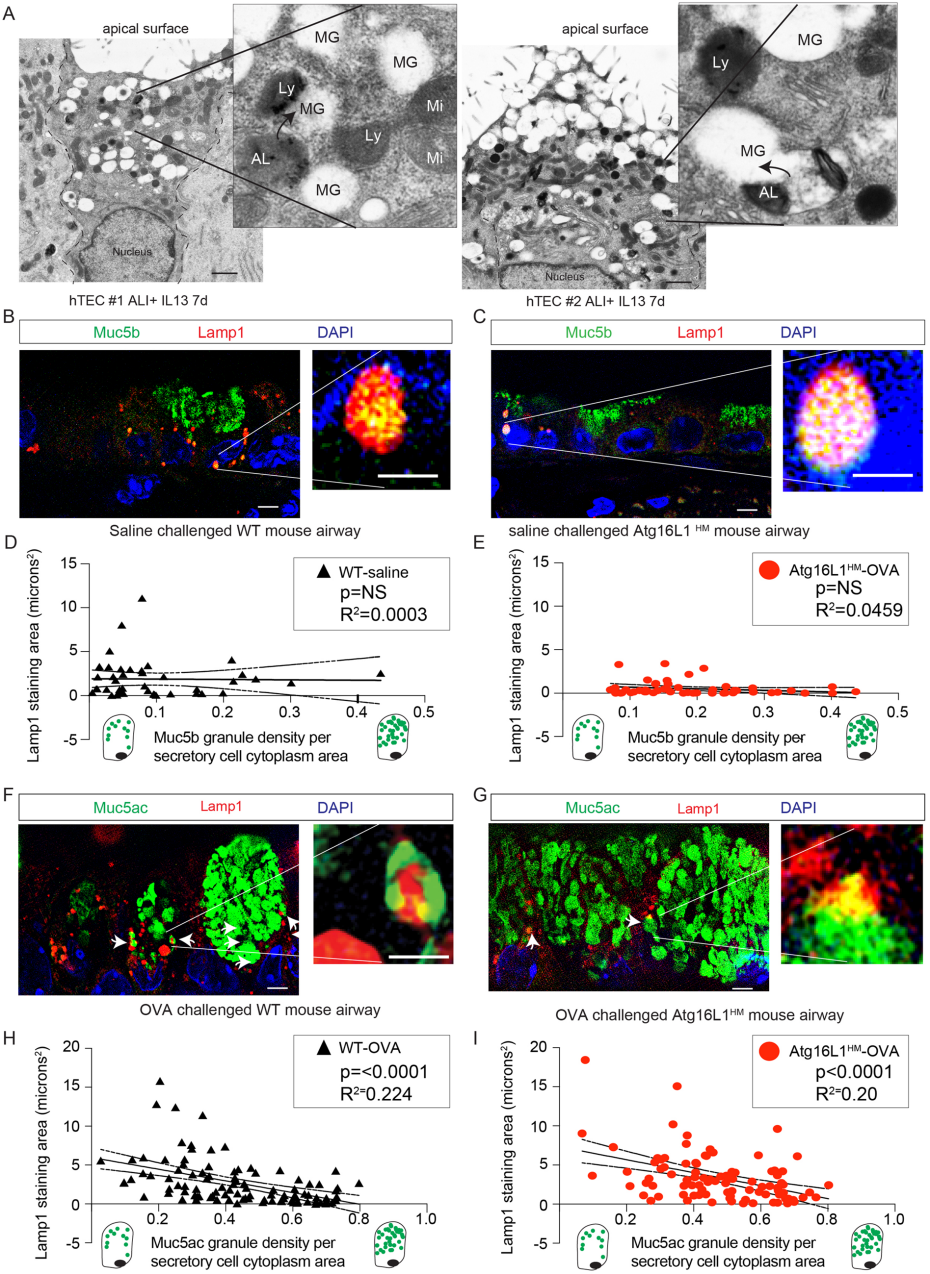
Airway secretory cells are enriched with Lamp1 associated lysosomes. (**A**) Representative transmission electron microscopy (TEM) images from human AECs treated with 7d IL-13 (10 ng/mL) under ALI conditions. Each image is from a different secretory cell from 2 unique AEC donors. Scale bar = 1 micron. Ly: lysosome, MG: Mucin granule, AL: autolysosome with double membrane, Mi: mitochondria. Arrows point to autolysosomes or lysosomes merging with mucin granules. (**B**, **C**) Representative images of saline challenged wildtype (WT) and Atg16L1^HM^ mouse airways with Muc5b staining (green) and Lamp1 (red). Scale bar = 5 microns. Nuclei are counterstained with DAPI. Image insets with scale bar of 1 micron. Linear correlation of Lamp1 staining intensity plotted against Muc5b granular density relative to secretory cell cytoplasm for WT (**D**) and Atg16L1^HM^ (**E**) mice. N = 40 and 50 mucous cells from 3 different mice WT and Atg16L1^HM^ per group. Representative image of OVA-challenged WT (**F**) and Atg16L1^HM^ (**G**) mouse airways with lectin UEA-1 for Muc5ac staining (green) and Lamp1 (red). Scale bar = 5 microns. DAPI for nuclear staining. Linear correlation of Lamp1 staining intensity plotted against lectin UEA-1 mucin granular density relative to secretory cell cytoplasm for WT (**H**) and Atg16L1^HM^ (**I**) mice. N = 100 mucous secretory cells from 4 different mice per WT and Atg16L1^HM^ group.

The autophagosome-lysosome pathway provides a means for protein degradation of proteins, aggregates^48^, and organelles^5,12,49–52^. We reasoned that if the mucin granules were being eliminated in the autophagosome-lysosome, we would find abundant lysosomes in close proximity with mucin granules. Transmission electron microscopy (TEM) images from primary human airway epithelial cells (AECs) treated with IL-13 for 7 days under ALI conditions revealed numerous lucent granules consistent with mucin granules^21,41,53^ concentrated at the apical pole of secretory cells. We also observed numerous vesicles with electron dense granules consistent with lysosomes^2,54–56^ that frequently appeared to communicate directly with mucin granules (Fig. 4A). There were also several examples of autolysosomes with remnants of the autophagosome double membrane and electron dense granules merging with mucin granules (insets of Fig. 4A). To verify the location of lysosomes in mucous cells, we next examined immunostaining of lysosomal membrane marker, Lamp1, by super resolution structured illumination microscopy (SR-SIM) in airways of WT and Atg16L1^HM^ mice challenged with saline or OVA. In the absence of MM, there are relatively few detectable mucous cells with only Muc5b positive granules. In saline challenged mice, Lamp1-positive lysosomes were more often located along the border of the nucleus in secretory cells and occasionally co-localized with Muc5b granules near the nucleus (Fig. 4B–E). OVA induced MM causes robust production of Muc5ac granules easily detectable in the axial airway epithelium. In OVA stimulated WT and Atg16L1^HM^ mice, we observed larger and more abundant Lamp1 positive lysosomes concentrated both along the border of the nucleus and the cytoplasm in close proximity to mucin granules (Fig. 4F,G). We also observed a strong concentration of the lysosomal membrane marker, Lamp2A, surrounding mucin granules. However, there was little staining of either Lamp1 or Lamp2A in ciliated cells in OVA challenged mouse airways (Fig. S3A–C). After a thorough review of the OVA-challenged mouse airways, we observed that larger well-formed secretory cells with higher Muc5ac granular density per cytoplasm area often had few Lamp1 positive lysosomes while secretory cells with lower mucin granular density had more abundant Lamp1-labeled lysosomes that were adjacent to mucin granules (Fig. 4H). The inverse relationship of Lamp1 staining and mucin granule density was also observed both in the WT and Atg16L1^HM^ OVA-challenged mice (Fig. 4I) but not in naïve WT or Atg16L1^HM^ mice (Fig. 4D,E). The variation in lysosomal staining suggests that there is a dynamic population of mucous cells with distinct stages and lysosomes were abundant only in later stages.

### Persistent retention of airway Muc5ac and Muc5b in autophagy deficient mice during resolution of mucous cell metaplasia

**Figure 5:**
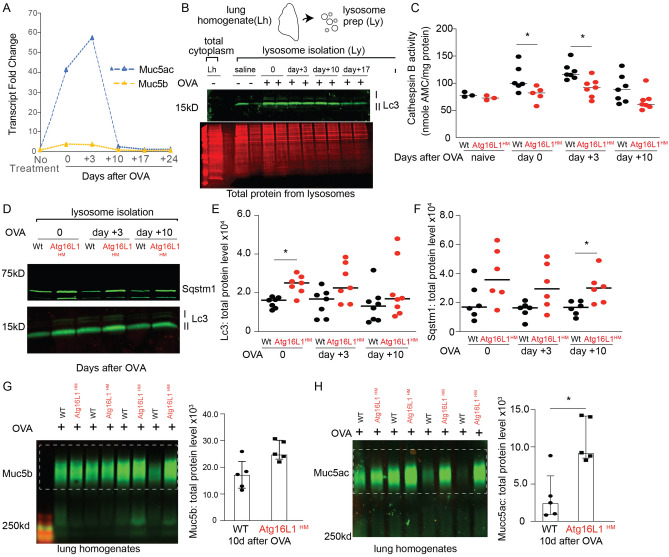
Atg16L1 deficient mice have slower resolution of mucous metaplasia following Type 2 airway inflammation. (**A**) Muc5ac and Muc5b expression levels were measured by qRT-PCR from a representative WT mouse lung homogenate in a time course experiment at 0, 3, 10, 17, and 24 days after the last OVA treatment. Data reported as fold change vs. non-treated mice (N = 4 for timepoints 0, 3, and 10 and n = 1 mouse at day 17 and 24). **(B**) Representative Lc3 immunoblot from whole lung homogenates (Lh) or after lysosome isolation (Ly) was performed from WT mouse lung homogenates ± OVA sensitization and challenge and then day 0, 3 and 10 from last OVA challenge with total protein stain shown below. (n = 2 per group) (**C**) Cathepsin B proteolytic enzyme activity was measured from isolated lysosome fractions (Ly) from mouse lung homogenates ± OVA. Enzyme activity normalized by total protein (n = 2 per group for naïve mice and n = 6 per group at day 0 and 7 per group at days 3 and 10 after OVA. (**D**) Representative Lc3 and Sqstm1 immunoblots from WT and Atg16L1^HM^ mouse lung lysosomes at day 0,3, and 10 from last OVA challenge. Quantification of Lc3 II (**E**) and Sqstm1 (**F**) according to time from last OVA challenge from WT and Atg16L1^HM^ lysosomal isolates (N = 7 mice per group). (**G**, **H**) Representative immunoblots of Muc5b and Muc5ac by lectin Uea-1 from WT and Atg16L1^HM^ mouse lung homogenates at 10 days after last OVA challenge with corresponding quantification. Mucin band density values normalized to total protein levels. (n = 5 mice per group). Graphs show scatter plots with median bar for cathepsin activity and Lc3/Sqstm1 western blotting and scatter plots with median bar and interquartile range for mucin blots. Significant difference by Mann–Whitney * for genotype difference of cathepsin B activity, Lc3, Sqstm1 immunoblots, and mucin blots in parts (**G**) and (**H**).

Little is known about the factors that regulate resolution of mucous metaplasia. It is possible that all the mucin granules are released into the airway. Alternatively, some of the granules may be eliminated by the autophagosome-lysosome pathway. Given the role of autophagy in recycling of cytoplasmic proteins, we next examined whether autophagy deficient mice had defects in resolution of mucous metaplasia related to reduced lysosomal mucin breakdown. Mice given OVA sensitization and challenge had a substantial increase in Muc5ac production by qPCR that decreased by day 10 after last OVA challenge to near baseline (Fig. 5A). We previously reported that levels of the autophagosome marker, LC3B II, remain elevated in human AECs after withdrawal of IL-13 or IL-4 from culture media suggesting persistent autophagy activity during resolution of mucous metaplasia^8^. We therefore examined isolated lysosomes derived from mouse lung homogenates following OVA stimulation. These lysosomal isolates were highly enriched for Lc3 II compared to the cytosol and levels peaked at days 0 to 3 after the last OVA challenge before falling at day + 10 and 17 (Fig. 5B). Next, we measured lysosome activity of cathepsins B, which is a cysteine protease representative of lysosome enzyme activity^57,58^. Cathepsin B proteolytic activity increased with OVA challenge but was highest on the 3rd day after the last OVA challenge (day 3), and then declined by day 10. Furthermore, there was lower proteolytic enzyme activity in the lysosomes from Atg16L1^HM^ mice at all time points (Fig. 5C). We also found that Atg16L1^HM^ mice had higher levels of Lc3B II and Sqstm1 from lysosomal isolates at all time points (Fig. 5D–F), which signifies that the Atg16L1^HM^ mice have reduced autophagosome-lysosome activity.

**Figure 6:**
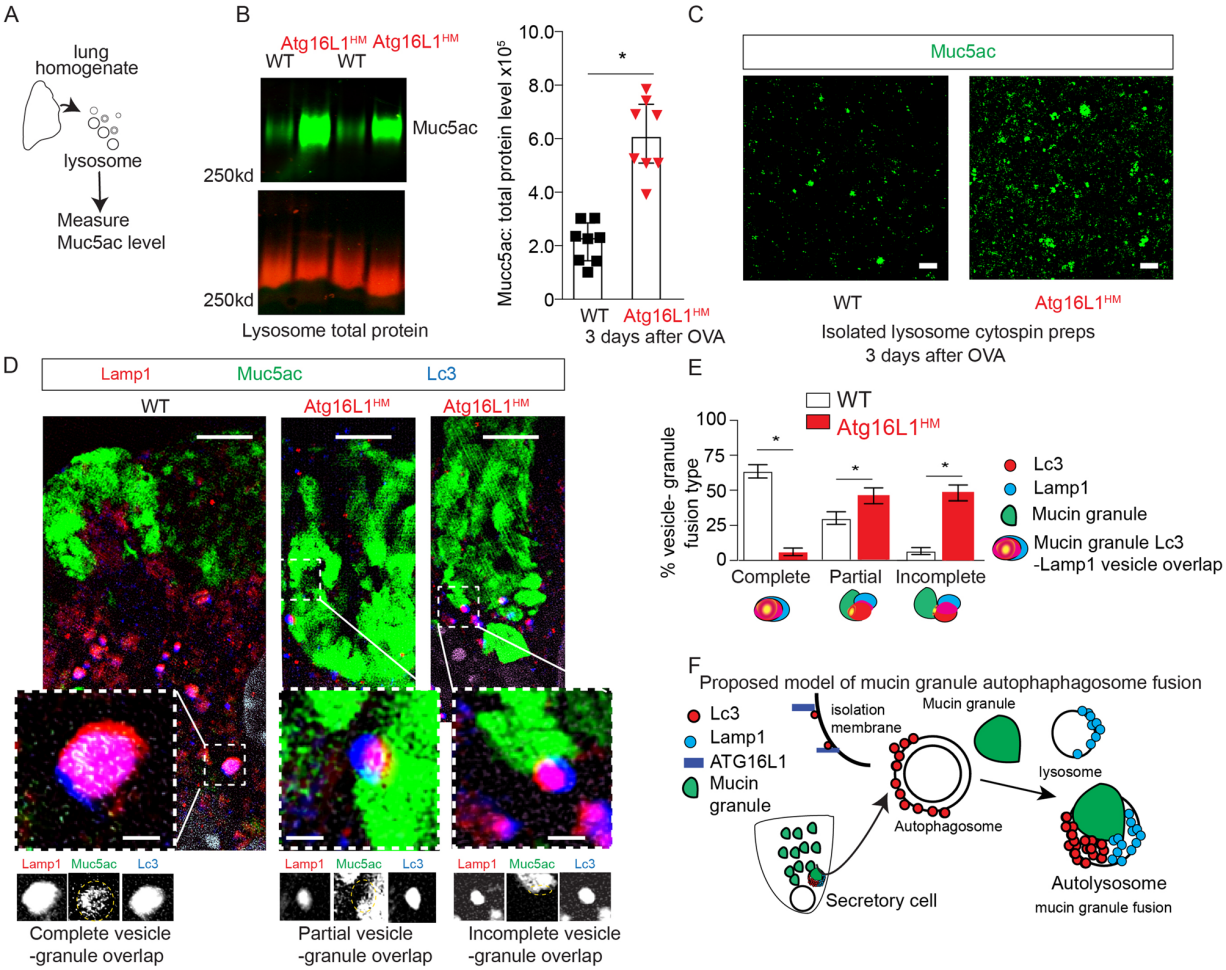
Atg16L1 deficient mice have increased partial or incomplete fusion of Lc3, Lamp1- labeled vesicles with mucin granules at day 3 of resolution from OVA-mediated mucous metaplasia. (**A**) Lysosome were purified from WT and Atg16L1^HM^ mouse lungs at day 3 of resolution from OVA-mediated mucous metaplasia as described in Fig. 5. (**B**) Representative immunoblot for Muc5ac detected by lectin UEA-1 from WT and Atg16L1^HM^ mouse lung lysosome preparations with corresponding quantification normalized to total protein (n = 8 mice for each group). (**C**) Representative SR-SIM images of cytospins on glass slides from lysosome preparations of WT and Atg16L1^HM^ mouse lungs stained by Muc5ac using lectin UEA-1 (green). (N = 7 images from 4 different mice for both WT and Atg16L1HM groups). Scale bar = 2 microns. (**D**) Representative SR-SIM images from WT and Atg16L1^HM^ mouse airways at day 3 of resolution from OVA-mediated mucous metaplasia with LC3 (blue), Lamp1 (red), and Muc5ac detected by lectin UEA-1 (green). Scale bar = 5 microns. Insets below show each magnified image of fusion event and have scale bar = 1 micron. Individual channels are shown below inset with gold dashed circle showing area of overlap. (**E**) Quantification fusion events among Lc3 and Lamp1 vesicles and mucin granules. N = 3 Atg16L1HM mice with 26 distinct secretory cells and 81 fusion events. N = 5 WT mice with 24 distinct secretory cells with 104 fusion events. Significant difference by Mann–Whitney * for genotype difference of Muc5ac immunoblots in (**B**) and unpaired Student T-test for fusion events between WT and Atg16L1^HM^ mice (**E**). (**F**) Proposed model of mucin granule fusion with Lc3 and Lamp1 positive vesicles in airway secretory cells as a means to eliminate excess mucin granules during resolution.

We then focused our attention on the impact of reduced autophagosome-lysosome activity in Atg16L1 deficient mice during the resolution phase after the last OVA challenge on the remaining intracellular mucin granules. We found a significantly greater amount of Muc5ac and Muc5b by western blot in the lungs of Atg16L1^HM^ mice (Fig. 5G–H). A similar finding was observed in mice 10 days after the last of 3 doses of intra-nasal IL-33 (Fig. S4). To determine why there is more intracellular mucin proteins during resolution, we turned our attention to the autophagosome pathway as a means of controlling intracellular mucin granule content. Since lysosomal cathepsin proteolytic activity and Lc3 II levels (Fig. 5C–E) were greatest at day 3 of resolution after OVA-mediated mucous metaplasia, we measured Muc5ac by lectin Uea-1 from the lysosome enriched fraction of lung homogenates at this timepoint. We found a robust increase in Muc5ac protein levels by immunoblot from lysosome preparations from Atg16L1^HM^ lungs (Figs. 6A,B), S6) Cytospins from lysosomal preparations revealed this mucin signal consisted predominately of 0.5 to 1 micron size granules (Fig. S6). This finding was consistent with our earlier findings of higher protein levels of Lc3 II and Sqstm1 in Atg16L1^HM^ lysosomal preparations (Fig. 5D–E). We then undertook to examine autophagosome and lysosome fusion events by immunostaining with SR-SIM microscopy using Lc3 and Lamp1 antibodies respectively. We observed frequent complete fusion of mucin granules with Lc3 and Lamp1 vesicles consistent with autolysosomes (Fig. 6D). As with earlier results (Fig. 4B–I), most of mucin granules that fused with Lc3 and Lamp1 labeled autolysosomes were adjacent to nucleus and not near the apical surface. Interestingly, while there is complete fusion of Lc3/ Lamp1-labeled vesicles and mucin granules in WT mucous secretory cells, we found that in Atg16L1^HM^ mucous secretory cells, Lc3/Lamp1 labeled vesicles demonstrated partial or incomplete fusion with each other and with mucin granules (Fig. 6E). These findings suggests that the mucin granules near the basolateral pole of the mucous secretory cells fuse with autolysosome stage vesicles rather than being first engulfed by autophagosomes prior to lysosome fusion (Fig. 6F). Atg16L1^HM^ mice had fewer complete fusion events and was associated with increased protein levels of autophagosome targets Lc3 II and Sqstm1 (Fig. 5D–F), and mucin granules (Fig. 6A–E). Therefore, during resolution of mucous metaplasia, certain mucin granules are eliminated by fusion with autophagosome system rather than apically secreted. We next sought to determine whether intracellular mucins levels can be reduced by activating the autophagy pathway exogenously.

### Activation of autophagy by mTOR inhibition reduces intracellular MUC5AC protein levels

**Figure 7:**
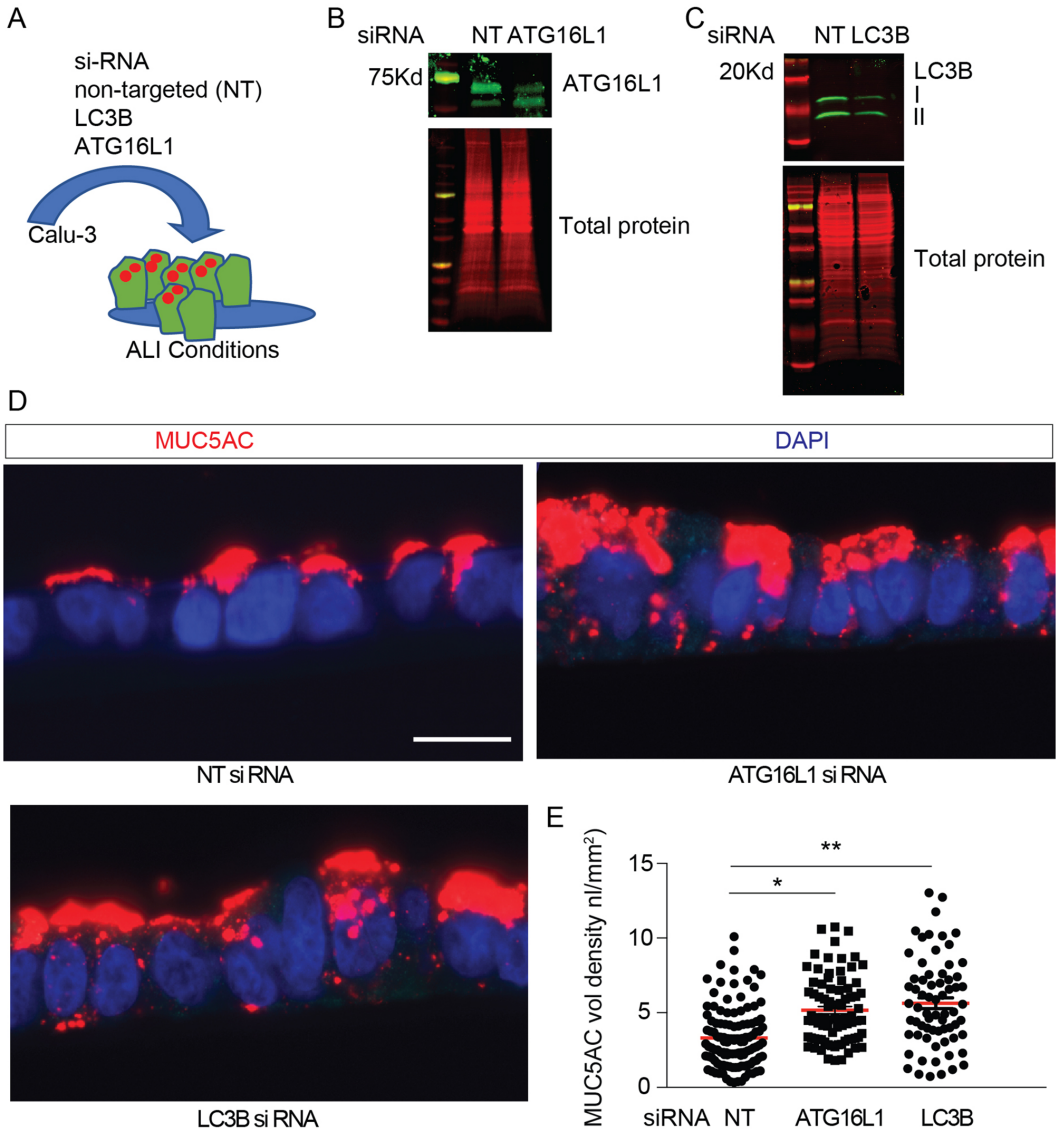
Autophagy deficient airway epithelial cells have increased intracellular MUC5AC granules. (**A**) Schematic for targeted depletion of ATG16L1 and LC3B levels in Calu-3 cells under ALI culture conditions. Representative immunoblots for Calu-3 cells transfected with non-targeted (NT), ATG16L1 siRNA (**B**) or LC3B siRNA (**C**). (**D**) Representative immunostaining using mouse anti-MUC5AC in Calu-3 cells transfected with non-targeted control (NT), LC3B, or ATG16L1 siRNA and corresponding quantification (**E**). Scale bar = 20 microns. (n = 3 experiments with 10–20 microscopic images per sample). Significant difference by ANOVA with Tukey’s post-hoc comparisons are noted by * NT vs Atg16L1 siRNA treatment difference or ** for NT vs. LC3B siRNA differences. Graphs show scatter plots with median bar for mucin immunostaining.

Calu-3 cells, which polarize and produce MUC5AC in granules^24,59,60^, were transfected with ATG16L1 or LC3B siRNA (Fig. 7A–C). Calu3 cells are an ideal model for monitoring degradation in vitro, because as cancer-derived airway epithelial cells, they accumulate mucin granules but don’t secrete in response to ATP-mediated signaling^24,60^. We found increased intracellular MUC5AC by immunostaining in the ATG16L1 or LC3B deficient Calu-3 cells (Fig. 7D,E). This indicates that MUC5AC is a target of the autophagosome-lysosome pathway and agrees with our earlier work that ATG5-deficient IL-13-challenged human AECs had increased intracellular MUC5AC^7^.

**Figure 8:**
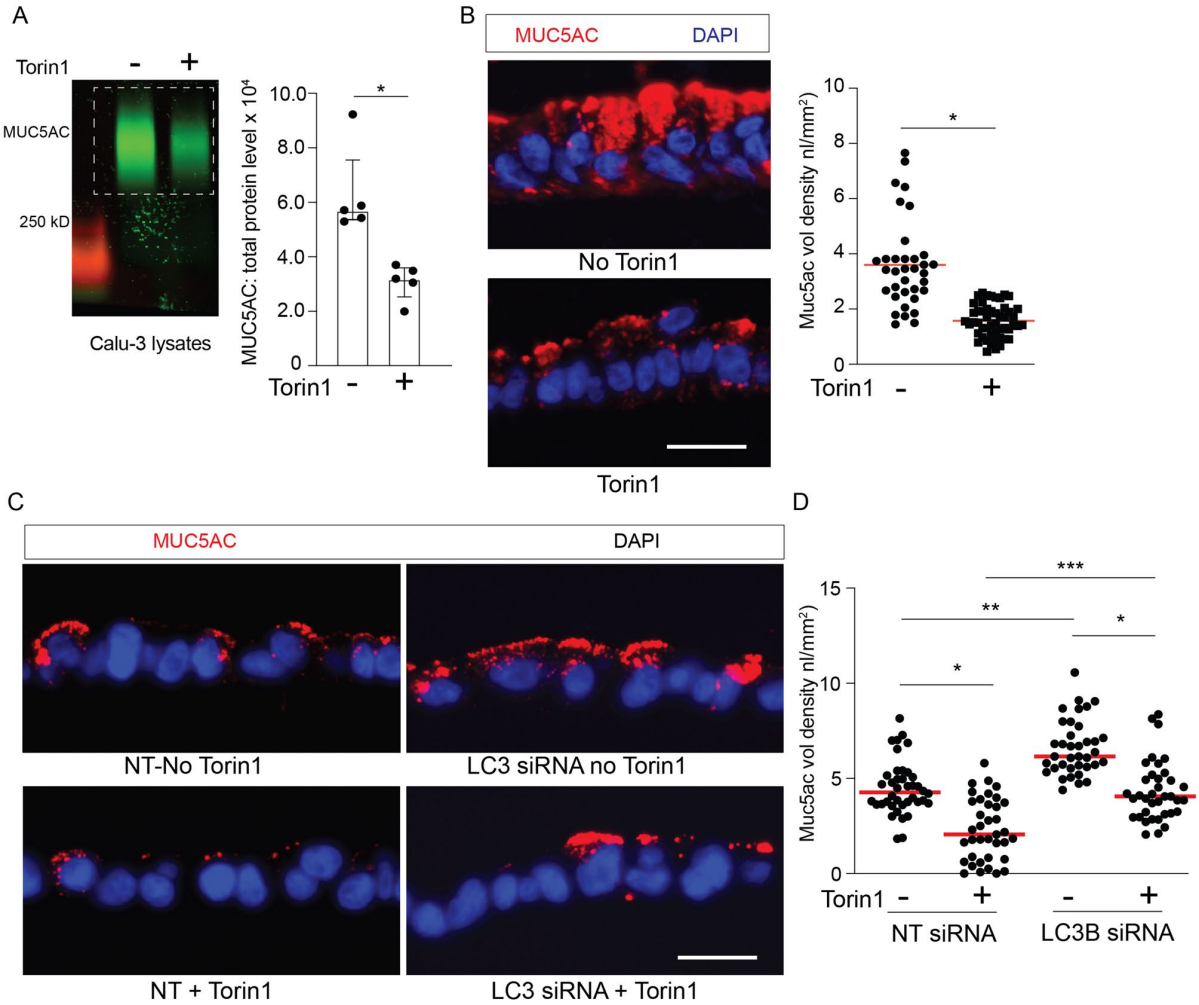
Activation of autophagy leads to decreased intracellular MUC5AC granules in human airway epithelial cells. (**A**) Representative immunoblot of MUC5AC from cell homogenates from Calu-3 treated ± torin1 (10 μM) for 18 h with corresponding quantification by total protein levels (N = 7 per group). (**B**) Representative MUC5AC immunostaining in Calu-3 cells under ALI conditions ± torin1 treatment. Scale bar = 25microns. Corresponding quantification of MUC5AC staining volume density. N = 3 experiments with 10–20 microscope images taken per sample. (**C**) Representative MUC5AC immunostaining of Calu-3 transfected with NT or LC3B siRNA and treated ± Torin1 (10 μM) for 18 h with corresponding quantification (**D**) of MUC5AC volume density. Scale bar = 25microns. N = 2 experiments with 20 microscope images taken per sample. Graphs show scatter plots with median bar for mucin immunostaining and scatter plots with median bar and interquartile range for mucin blots. Significant difference by Mann–Whitney test with * for torin1 difference for MUC5AC blot in part (**A**) and unpaired T-test for MUC5AC immunostaining in part **B**. For quantification of parts (**C**, **D**) significant difference by ANOVA with Tukey’s post-hoc comparisons are noted by * for torin1 treatment difference or ** and *** for siRNA differences.

**Figure 9:**
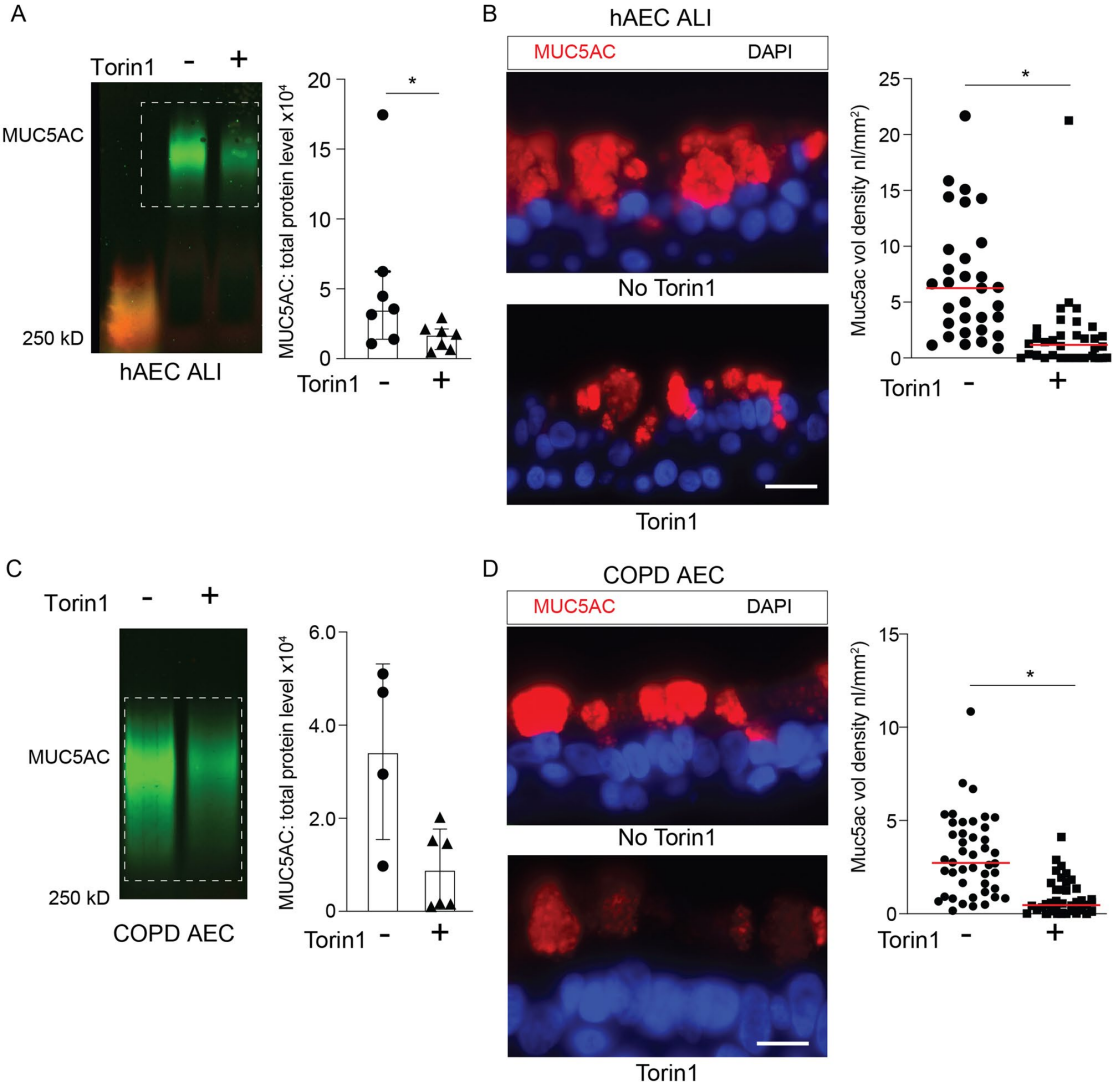
Activation of autophagy leads to decreased intracellular MUC5AC in human airway epithelial cells. (**A**) Representative immunoblot of MUC5AC from cell homogenates from human non-diseased AECs under ALI conditions treated with torin1 (10 μM) for 18 h with corresponding quantification by total protein levels (N = 5). (**B**) Representative MUC5AC immunostaining in human AECs under ALI conditions ± torin1 treatment (10 μM) for 18 h. Corresponding quantification of MUC5AC volume density. N = 2 experiments with 20 microscope images taken per sample. (**C**) Representative immunoblot for MUC5AC levels from cell lysates from human AECs under ALI condition derived from COPD explant airways. (n = 4 CTL and 6 torin1 treated inserts from 2 different lung explant donors) along with corresponding quantification to total protein levels. (**D**) Representative MUC5AC immunostaining in human COPD AECs under ALI conditions ± torin1 treatment (10 μM) for 18 h. N = 3 inserts from two different COPD donors with 12 microscope images taken per sample. Scale bar = 20 microns for (**B**) and (**D**). Graphs show scatter plots with median bar for mucin immunostaining and scatter plots with median bar and interquartile range for mucin blots. Significant difference by Mann Whitney test for mucin blot data in parts (**A**) and (**C**). Significant difference by unpaired T-test with * for torin1 difference for immunostaining data in parts (**B**) and (**D**).

We next asked if activation of autophagy by mTOR inhibition influenced intracellular MUC5AC levels, since Torin1 specifically inhibits mTORC1 and mTORC2 kinase activity in mammalian cells and leads to activation of autophagy^61^. We have previously shown in primary human airway epithelial cells (AECs) that torin1 increases autophagy-mediated degradation with increased LC3 II and decreased LC3 I^8^. In a similar fashion, Calu-3 cells under ALI conditions treated with torin1 for 18 h had increased LC3 II and reduced LC3 I protein levels and decreased phosphorylated mTOR (Fig. S5A,B). Torin1 did not lead to changes in MUC5AC gene expression (Fig. S5C), rather Torin1 reduced intracellular MUC5AC protein levels detected by mucin immunoblotting and immunostaining (Fig. 7A,B). The effect of Torin1-mediated reduction in intracellular MUC5AC granules was mitigated when LC3B was depleted by siRNA (Fig. 8A–D). We next looked at human AECs under ALI conditions. There was a significant decrease in MUC5AC levels in torin1 treated human AECs (Fig. 9A,B) and in COPD derived AECs (Fig. 9C,D). We measured the secreted MUC5AC from Calu-3 cells or human AECs under ALI conditions. Autophagy activation by mTOR inhibition with Torin1 led to a decrease in secreted MUC5AC consistent with the cell lysate findings and showing that Torin1 did not induce mucin secretion (Fig. S5D–F). These findings indicate that intracellular MUC5AC granules can be reduced by the activating the autophagosome-lysosome pathway.

## Discussion

Here, we show for the first time, that the autophagy-lysosome activity is essential for removing excess mucin granules during resolution of mucous metaplasia. This difference was not due to defects in baseline or stimulated secretion. Instead, we propose that autophagy-deficient mice had reduced ability to eliminate intracellular mucin granules. In support of this new paradigm, we found that airway mucous cells are enriched in Lc3 vesicles and Lamp1-2-labeled lysosomes that closely approximate or engulf mucin granules. The Atg16L1^HM^ mouse model has reduced autophagosome-lysosome activity and fusion events as evidenced by higher lysosomal specific Sqstm1 and Lc3 II at all timepoints during resolution, higher Muc5ac from isolated lysosomes and reduced Lc3, Lamp1 vesicle-mucin granule fusion. Interestingly, we did not observe a difference between WT and Atg16L1^HM^ mice in the association of abundant Lc3-Lamp1 vesicles and mucin granules primarily in secretory cells with fewer mucin granules nor in the distribution of Lc3-Lamp1 vesicles near the nucleus. Rather, Atg16L1 deficient mice had reduced Lc3, Lamp1 vesicle-mucin granule fusion events. Correspondingly, Atg16L1 deficient mice had reduced flux and showed a delay in resolution of mucous metaplasia.

Inflammatory insults, such as OVA or IL-33, lead to massive increases in Muc5ac protein synthesis, ER stress^62,63^, and oxidative stress^8,64^. Our findings suggest that airway mucous cells utilize autophagosome-lysosome activity to reduce the burden of excessive mucin granules to restore cellular homeostasis after the inflammatory insult is over. It may seem counterintuitive for mucous cells to provide an alternative fate for secretory proteins. However, this elimination pathway likely is a situational phenomenon reflecting changing cellular metabolism after loss of inflammatory signals. As inflammation reduces, the drive to produce and secrete large numbers of mucin granules decreases. This is why we mainly observed basolateral mucin granules near the nucleus fusing with Lc3/Lamp1-labeled vesicles. We hypothesize that mucous cells can easily secrete apical granules near the luminal surface as inflammatory stimuli wane and while deeper mucin granules could be targeted for degradation and recycling of amino acids. While our data shows clear fusion events among Lc3-Lamp1 vesicles and mucin granules that are reduced in Atg16L1^HM^ mice, we have not shown biochemical evidence of mucin glycoprotein breakdown products in the lysosome. Ongoing studies in the lab seek to confirm the Lc3-Lamp1 vesicle as a functional autolysosome, address the molecular mechanism of Lc3-Lamp1 vesicle-mucin granule fusion and identify mucin protein fragment in the lysosome from WT and autophagy deficient mice. Finally, we believe that our findings are not specific to Type 2 inflammation, as on-going studies in the lab suggest this is a broad phenomenon related to mucous metaplasia and resolution.

We, and others, have reported that autophagy is activated in the airway epithelium of Type 2 inflammation disease models^7–9^. These observations are largely based on the finding of increased LC3B II in the presence of autophagosome-lysosome inhibitors by immunoblot or immunostaining from mouse lung homogenates or airway epithelium. However, careful interpretation of our earlier data^7,8^ suggests that while the number of autophagosome structures is increased during airway inflammation, autophagosome flux or the proteolysis of the contents of the autophagosome is also, at least, partially inhibited. Indeed, our data from isolated mouse lung lysosomes shows elevated Lc3 II and Sqstm1 levels during the early resolution period that subsequently decrease over time. Collectively, our findings support a biphasic autophagy response with autophagy activation in response to external stimuli during inflammation and autophagy consummation during resolution with fusion to the lysosome and subsequent proteolysis. Pathways that regulate lysosomal biogenesis, function, and degradation (lysophagy) in the airway epithelium are not well understood. Here, we show that lysosomes are easily identified in the basal region of mucous cells and closely approximate mucin granules. Future studies are needed to elucidate the signals that regulate the formation, function, and degradation of lysosomes and how this relates to the resolution of mucous metaplasia.

Previous studies using chloroquine have postulated that autophagy inhibition may be a beneficial strategy in muco-obstructive airway disease^9^. Others have found that cell specific knockout of autophagy proteins in the hematopoietic system had no effect on IL-13 and IL-4 levels following House Dust Mite instillation^65^. Our data is a caution that autophagy action is cell and context dependent. Strategies to inhibit autophagy in the airway may have unintended consequences to prolong mucous metaplasia and prevent resolution. In fact, our data shows that activation of autophagy through mTOR inhibition leads to reduced intracellular and secreted levels of MUC5AC in non-diseased and COPD-derived human AECs. Previously, We have shown that AECs deficient in ATG5 and ATG14 had reduced IL-13-mediated apical washes for MUC5AC^7^. This finding agreed with similar data by collaborators in the gut epithelium, showing autophagy proteins are required for Muc2 secretion in the colon based on a ROS-mediated mechanism^66,67^. Airway mucin secretion relies on a baseline and stimulated secretion with distinct exocytic machinery that is unique compared to the gut epithelium. Our current findings examine both baseline and stimulated secretion and find no relationship with Atg16L1 deficiency. It is possible that autophagy regulatory proteins regulate secretion when inflammation and oxidant stress are highest in a non-canonical fashion (not related to autophagy-lysosomal degradation) while canonical autophagy eliminates excess mucin granules as a means to restore homeostasis during resolution.

This study provides evidence to alter the existing paradigm that only production and secretion regulate the level of intracellular mucin content. We propose that there is a third factor, degradation, that plays a role in resolving mucous metaplasia by eliminating mucin granules by autophagosome-lysosome action. Our findings offer a new potential therapeutic strategy to speed the resolution of mucous metaplasia in those with exacerbations of muco-obstructive lung diseases.

## Materials and methods

### Mice

C57BL/6J wild-type (WT) were purchased from The Jackson Laboratory (Bar Harbor, ME). Atg16 hypomorph (HM) mice with global deficiency of Atg16L1 gene on the same C57BL/6J background^42^ were obtained courtesy of Thaddeus Stappenbeck (Washington University, St Louis, MO). Male and female mice were randomly selected and utilized in equal numbers between 12 and 16 weeks of age for all experimental studies. Food and water were provided ad libitum. Each mouse was assigned a unique letter and number combination which was identify samples.

WT or Atg16L1^HM^ mice were lightly anesthetized by isoflurane inhalation before intranasal inhalation of 50 μl of sterile saline or 1 μg of IL-33 daily for 5 days. Alternatively, mice were OVA-albumin sensitized by intraperitoneal injection × 3 over 7 days. After 7-day incubation period, mice were subsequently challenged by nebulization daily over 5 days as previously described^8^. To assess the role of Atg16L1 in the secretory response of intracellular mucins to ATP, a subset of WT and Atg16L1^HM^ mice on the last day of OVA challenge were placed in a plexiglass chamber and exposed to nebulized ATP (Sigma-Aldrich A6419) (100 mM diluted in sterile water) 30–60 min prior to sacrificing the animal as previously described^25,46^.

### Cell culture techniques

Mouse tracheal epithelial cells (mouse AECs) were isolated after pronase (Sigma-Aldrich, 9036-06-0) digestion of WT and Atg16L1^HM^ mouse tracheas and grown on supported membrane inserts (Corning #3460 and #3470) as previously described^68–70^. Mouse AECs were expanded (5–8 days) using DCI media containing: isobutyl methylxanthine (IBMX), final concentration 0.1 mM (Sigma, I5879), 8-bromoadenosine 3′,5′-cyclic monophosphate sodium (8-BAMS), final concentration 0.1 mM (Sigma, B7880), calcium chloride final concentration 1 mM (Sigma), L-glutamine final concentration in Advanced DMEM/F12 500 ml (Gibco#12634-010) and then fed with PneumaCult ALI media (Stemcell Technologies # Catalog #05001) under air liquid interface (ALI) conditions . Non-diseased human airway epithelial cells (AECs) were derived for culture from excess airway tissue donated for lung transplantation as previously described^7^. COPD AECs were derived by pronase digestion from COPD explanted lungs following transplantation. After expansion and antibiotics treatments, the COPD AECs were passaged on to supported inserts for ALI differentiation. After 5–7 days of expansion with BEGM media (Lonza #CC-3170), air–liquid interface (ALI) conditions were established and non-diseased and COPD AECs were fed with PneumaCult ALI media. Calu-3 epithelial cells were chosen for their ease in transfection and ability to polarize under ALI conditions and produce mucin MUC5AC granules^59,60^. Calu-3 cells obtained from ATCC (#HTB-55) between passage 25–35, were cultured in Eagles Minimal Essential Media (ATCC #30-2003) media plus 10% FBS on supported membranes as above for AECs. Cells were utilized 7–14 days after the establishment of ALI conditions. ATCC authenticated the identity of Calu-3 used in these experiments.

### Immunoblotting

Cell lysates cultured from Calu-3 cells or mouse AECs were isolated in RIPA buffer with protease inhibitor on ice for 20 min with brief water bath sonication. Cell lysates for mTOR and P-mTOR detection were lysed in a NP-40 buffer: 50 mM HEPES, 0.5% NP40, 2.5 mM EDTA, 50 mM NACL, 10 mM sodium pyrophosphate, 50 mM sodium fluoride, and protease inhibitor cocktail. Samples underwent electrophoresis on a gradient 4–20% gradient acrylamide gel and then were transferred on PDVF membranes over 2 h. The blot was rinsed with TBS with 0.1% tween and stained with REVERT Total Protein Stain with conjugated 700 nm fluorochrome (LI-COR, Lincoln, NE) for 5 min. Blots were imaged using an Odyssey CLx Imager (LI-COR) for total protein detection. Membranes were blocked with either 5% BSA in TBS with 0.1% Tween for or 5% non-fat milk for one hour at room temperature and then incubated with primary antibody overnight at 4 °C in the diluted appropriate blocking buffer: Scgb1a1 1:500 vol:vol, (Seven Hills Bioreagents #WRAB-3950), Atg16L1 1:1000 vol:vol (Cell Signaling #D6D5), total mTOR and phosphorylated (Serine 2448) mTOR (1:1000 vol:vol (Cell Signaling #2972S and #2971S). After washing with TBS and 0.1% tween, the IRDye 800 nm CW goat anti-rabbit (Li-Cor 926-32211) at a 1:10,000 dilution in blocking buffer was added to the blot and incubated at room temperature for 1 h, and levels were normalized to total protein levels. Calu-3 total cell lysates were solubilized for LC3 immunoblot and run on a 15% polyacrylamide gel for transfer onto PDFM membranes as previously described^7,8^. LC3 was detected by rabbit polyclonal LC3B II 1:500 vol:vol (Sigma-Aldrich L7543). Levels were normalized either to ACTIN or total protein levels.

Mouse lungs were prepared for immunoblotting as previously described^8^. Briefly following euthanasia, the right heart was cannulated, and sterile PBS with heparin sulfate (0.67Units/mL) was infused to remove blood from the pulmonary vasculature. Lungs were homogenized in RIPA buffer with protease inhibitor using a tissue dissociator (GentleMacs Dissociator Miltenyi Biotec). Homogenates were then centrifuged at 10,000 rpm and 4 °C and the supernatants were collected and prepared for either Lc3 or Atg16L1 detection as described above.

### Mucin immunoblots

After euthanasia, excised mouse lungs were snap frozen in liquid nitrogen. Lungs were then later homogenized in PBS with protease inhibitors (Roche, Mannheim, Germany) using a gentleMACS dissociator (Miltenyi Biotec GmbH, Bergisch Gladbach, Germany). Subsequently the lysates were centrifuged for 30 min at 16,000 rpm at 4 °C. Lung homogenate supernatants were then collected and quantified. Loading buffer (containing 5 mM dithiothreitol (DTT) for Muc5b detection or no DTT for Muc5ac by UEA-1 detection. Electrophoresis was performed on a 0.8% agarose gel. The gel was incubated in 10 mM DTT saline sodium citrate (SCC) buffer (3.0 M sodium chloride, 0.3 M sodium citrate, pH 7.0) at room temperature for 20 min then rinsed in water for Muc5b detection. Gels with Muc5ac by UEA-1 detection were rinsed with SCC buffer without DTT and proceeded directly to transfer. The protein was then transferred onto a 0.45-micron nitrocellulose membrane (BioRad, Germany) using a vacuum Blotter (Model 785, BioRad). Blots were rinsed with TBS and blocked with TBS with 5% non-fat milk for 1 h at room temperature and then incubated with mouse monoclonal antibody for Muc5b (1:500 vol:vol) or lectin UEA-1 L8146 1 mg/mL (1:1000 vol:vol) (Sigma-Aldrich) overnight at 4 °C. IRDye 800 nm CW Goat anti-Mouse (Li-Cor) at a 1:10,000 dilution for Muc5b or rabbit anti-lectin UEA-1 (Sigma, U4754) at a 1:1,000 dilution in blocking buffer to detect Muc5ac was added to the mucin blot and incubated at room temperature for 1 h. IRDye 800 nm CW goat anti-rabbit (Li-Cor) at a 1:10,000 dilution in blocking buffer was added to the UEA1/Muc5ac blot and incubated at room temperature for 1 h. Mucin blots were normalized for quantification using total protein levels as previously described^46^.

### Assessment of airway epithelial immunostaining

Following euthanasia and lung perfusion, the whole lungs were then excised and slowly inflated with 10% formalin and hung under 20 cm of H20 pressure for 24 h. Fixed lung tissue was embedded in paraffin and 5-micron lung sections were cut. Antibody and lectin-specific staining and quantification was performed on embedded lung tissue slides as previously described^46^. Staining for lysosomal membrane markers Lamp1 and Lamp2A was done with rabbit polyclonal antibodies 1:500 vol:vol (Cell Signaling; #D2D11 and ThermoFischer; #51-2200 respectively). Staining for Lc3b was done with mouse monoclonal antibody (Enzo #5F10). Staining for cilia was performed using mouse monoclonal antibody to acetylated alpha tubulin 1:1000 vol:vol (Cell Signaling #11H10). Secondary fluorophore-labeled donkey secondary, species-specific antibodies were Alexa Fluor 488 or 555 (Life Technologies; #A-31570, #A21202, #A21206, #A31572). Nuclear counterstaining of DNA was obtained with 4′,6 diamidino-2-phenylindole (DAPI). Mouse airway or cultured AECs mucin volume density was quantified as described previously^8,25^. Images were obtained using a Zeiss Axio Observer Z.1 microscope and analyzed by Zen software (Zeiss). Super resolution images were obtained using a Zeiss Axio Observer Z.1 microscope and analyzed by Zen software (Zeiss). Super resolution Structured Illumination Microscopy for Optical Section (SR-SIM) obtained images with 0.110-micron Z-stack slices by Zeiss PS.1 confocal microscope. For quantification of SR-SIM images of mucous cells and lysosomal markers, a single Z-stack was chosen that contained multiple mucous cells. A common image threshold was utilized for each fluorescent channel by ImageJ software. The amount of LAMP1 staining by image intensity using a common threshold was measured for each mucous cell and dichotomized for mucous cells with ≤ or > 50% Muc5ac staining area of the total mucous cell cytoplasm or ≤ or > 33% of Muc5b staining area of the total mucous cell cytoplasm. To characterize the type of fusion events among Lc3 and Lamp1 vesicles and mucin granules, we captured SR-SIM images as described above from WT and Atg16L1^HM^ mouse airways at day 3 of resolution from OVA-mediated mucous metaplasia. We utilized 0.2-micron Z-stack slides using a Zeiss PS.1 confocal microscope. Fusion events were characterized as complete if the mucin granule was complete within the autophagosome-lysosome borders. It was characterized as partial if the Lc3 and Lamp1 vesicles partially overlapped and partially merged with the mucin granule. It was characterized as incomplete if the Lc3 and Lamp1 vesicles only partially overlapped and only minimally merged with the mucin granule or not at all. Images were taken from 3 Atg16L^HM^ mice (26 secretory cells; 81 observed triple fusion events) and 5 WT mice (24 secretory cells; 104 observed triple fusion events). The percentage of each type of fusion event was calculated by the number of each type over the total number and compared between WT and Atg16L1^HM^ mice.

Mouse/human airway epithelial cells (AECs) and Calu-3 staining was performed in cross sections of paraffin embedded formalin inserts. First, AECs were embedded in warmed 37 °C 1% agarose. After cooling, the inserts were fixed with 10% formalin and embedding in paraffin. Cross sections from the inserts were then cut and fixed on slides for immunostaining as previously described^46^. Using the same antigen retrieval and blocking method described above for mouse lung sections, AECs were stained with (1:500 vol:vol dilution) mouse monoclonal antibody to MUC5AC (Thermo Fischer Scientific #MA5-12178) for human AECs and Calu3 cells. Mouse AECs and airways were stained for Muc5b by mouse monoclonal antibody 3E1^26,27^, Muc5ac by lectin UEA-1 L8146 1 mg/mL (1:1000 vol:vol) (Sigma-Aldrich) and by rabbit polyclonal UNC294 provided courtesy of Dr. Camille Ehre and rabbit antibody Scgb1a1 (Seven Hills BioReagents WRAB-3950).

### Electron microscopy

Cultured human AECs under ALI conditions treated for 7 days with IL-13 (10 ng/mL) were fixed with a solution containing 4% paraformaldehyde and 0.5% glutaraldehyde and processed for transmission electron microscopy as previously described^7^.

### Lysosomal isolation and enzymes activity

Mouse lungs were perfused with PBS and heparin as described above. Then one lung was collected for lysosome isolation (Minute Lysosome Isolation Kit; Invent Technologies; LY034) per manufacture’s protocol. Briefly, lungs were dissociated by forcing the tissues against the collection column for one minute and then incubated on ice for 5 min. A series of column centrifugations was used to enrich lysosomes from the crude cytoplasm. The final pellet lysed in 40 ml of denaturing protein SDS solubilization reagent (Invent Technologies; WA-009) and then quantified prior to immunoblot or protease activity assay. To determine lysosome protease activity from lysosomes of lungs challenged with OVA, Cathepsin activities were measured after incubating isolated lysosomes with cathepsin B and cathepsin L fluorogenic substrates as described earlier^58,71^. To measure mucin levels in the lysosome fraction, we proceed with lysosome isolation as described above. After the last elution, instead of using denaturing protein SDS solubilization reagent, the solution was sonicated as described above for mucin isolation from lung homogenates. We then proceeded as described above for mucin immunoblotting. To examine the contents of the lysosome isolation for mucin granules, lysosomes were isolated as previously described, except being resuspended in 500 uL of 1X PBS per right lung for the final step. Resuspended lysosomes were centrifuged at 500 rpm for 5 min onto glass slides, and air dried at least overnight. Slides were fixed for 10 min in 4% paraformaldehyde in 1X PBS, rinsed 3 times for 5 min in 1X PBS and then stained as previously described for Muc5ac using lectin UEA-1.

### RNA interference

ATG16L1, LC3B, or control, non-targeted (NT) siRNA sequences (Dharmacon On-Target Plus; #L-021033-01, # L-012846-00-0005, #D-001810-10-20) were transfected in Calu-3 on day 0 of culture on insets. The siRNA oligonucleotides (80 nM) were suspended in Opti-MEM 1 (25%; Life Technologies, #31985-070) and Lipofectamine RNAiMAX (Invitrogen; #13778-150) as previously described^8^. Depletion of ATGL16L1 and LC3B was verified on day 7 of ALI culture conditions by immunoblot analysis using antibodies to rabbit anti-ATG16L1 (1:1000 vol:vol Cell Signaling D6D5) or rabbit anti-LC3B (1:500 vol:vol Sigma-Aldrich L7543). Cells were then agarose embedded and formalin fixed for staining as described above or homogenates collected for mucin blot at ALI day 10.

### Autophagy induction and measurement of MUC5AC in Calu-3 and human AECs

To determine intracellular mucin levels in vitro, the apical surface was rinsed with 100 μl pre-warmed PBS lacking calcium and magnesium for 15 min at 37 oC a total of three times prior to the experiment. For autophagy induction assays, non-diseased or COPD derived AECs were treated with 10 μM torin1 (Sigma Aldrich #475991) and or vehicle control DMSO 1/1000 working dilution in the basolateral media and apical PBS (150 mL volume). For mucin collection, cells were collected in PBS with protease inhibitor, homogenized using probe sonication for 3 s, centrifuged for 30 min at 16,000 g at 4 °C, and quantified. Mucin immunoblotting was then continued as described above for mouse lung homogenates. Mouse monoclonal anti-MUC5AC (Thermo Fischer Scientific #MA5-12178) was used to detect MUC5AC from human AECs and Calu-3 cells. No DTT reducing agent was added to the loading buffer or transfer buffer for MUC5AC detection in human cells by 45M1 mouse antibody.

### PCR gene expression

RNA was isolated from whole mouse lung tissue or Calu-3 cells using the RNeasy Spin column kit (Qiagen #75144) then reverse transcribed using the cDNA Reverse Transcription Kit (Applied Biosystems #4308228) as previously described^46^. Gene expression was reported as fold change over WT-saline condition normalized to GAPDH in mouse lung tissues or OAZ1 for Calu-3 cells and calculated using the double delta CT (2∆∆CT) method as previously described^7,8,46^.

### Statistical analysis

Significant difference by unpaired T test or ANOVA with Tukey’s post-hoc comparisons are noted by * for treatment difference or ** for mouse genotype differences for normally distributed immunostaining data. Kruskal Wallis or Mann Whitney test was performed for non-parametric mucin blot data quantification.

### Study approval

All animal procedures were approved by the Institutional Animal Care and Use Committee of the University of Nebraska Medical Center. All mice were experiments were performed according to corresponding guidelines and regulations including ARRIVE guidelins^72,73^. Human AECs derived from (1) healthy lungs not suitable for transplant or from (2) COPD lung explants after transplant were isolated under protocols approved by the University of Nebraska Medical Center. Regarding the sources of tissue: The University of Nebraska Medical Center (UNMC) Division of Pulmonary, Critical Care, and Sleep Medicine maintains a tissue bank that stores lung tissue, bronchoalveolar lavage fluid (BALF), and blood samples from (1) de-identified non-diseased lungs and trachea not suitable for transplant accepted from the International Institute for the Advancement of Medicine (IIAM), the National Disease Research Interchange (NDRI) and Live On Nebraska. All 3 non-profit groups provide de-identified tissue for research. No identifiers are provided, and identifiers are not accessible from the repository. (2) Similarly, the UNMC tissue bank maintains tissue and blood from diseased lung explants from patients who undergo a lung transplantation at UNMC. This protocol is approved by the UNMC IRB.

## Supplementary Information

Supplementary Information.
