# Supplementary material for: Autophagy of mucin granules contributes to resolution of airway mucous metaplasia
Source: Sci Rep. 2021 Jun 22;11:13037. doi: 10.1038/s41598-021-91932-7 (PMC8219712; doi:10.1038/s41598-021-91932-7)
Supplement: Supplementary file 1 — Supplementary Information. [file 41598_2021_91932_MOESM1_ESM.docx]

**Title: Autophagy of mucin granules contributes to resolution of airway mucous metaplasia.**

**Authors**: Sweeter, JM^1^, Kudrna K^1^, Hunt, K^1^, Thomes, P^1^, Dickey, BF^2^, Brody SL^3^, Dickinson, JD*^1^

**Institutions**: 1) Pulmonary, Critical Care, Sleep and Allergy Division, Department of Internal Medicine, University of Nebraska Medical Center, Omaha, NE, USA. 2) Department of Pulmonary Medicine, MD Anderson Cancer Center, Houston, TX, USA. 3) Department of Medicine, Washington University School of Medicine, Saint Louis, MO, USA.

*Correspondence to: jdickins@unmc.edu

**Supplemental Data**

**
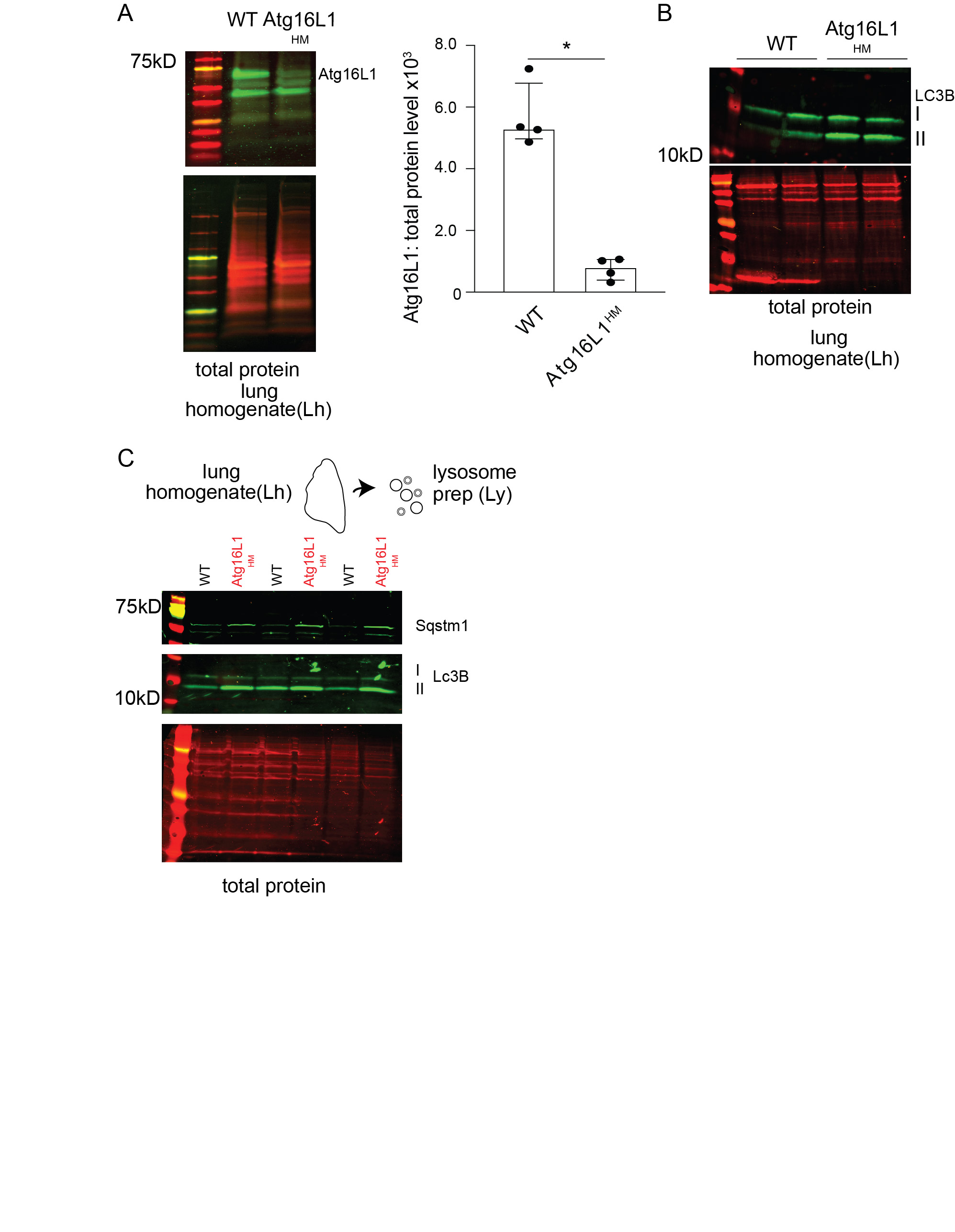
Figure Legend:**

**Figure S1**: **Atg16L1HM mice have reduced Atg16L1 and increased LC3II levels in lung homogenates and isolated lysosomes.**  (**A**) Representative immunoblots for Atg16L1 with corresponding quantification from lung homogenates in naïve WT and Atg16L1^HM^ mice (N=4). Atg16L1 values normalized by total protein levels. (**B**) Representative blot of LC3 levels in naïve WT and Atg16Hl1^HM^ mouse lungs (n=2). (**C**) Representative Lc3 and Sqstm1 immunoblots from isolated lysosomes (Ly) derived from lung homogenates of naïve WT and Atg16L1^HM^ mice. N=3 mice per group. Graphs show scatter plots with median bar and interquartile range for western blots. Mann Whitney test to detect significance difference in Atg16L1 lung levels in **A**.


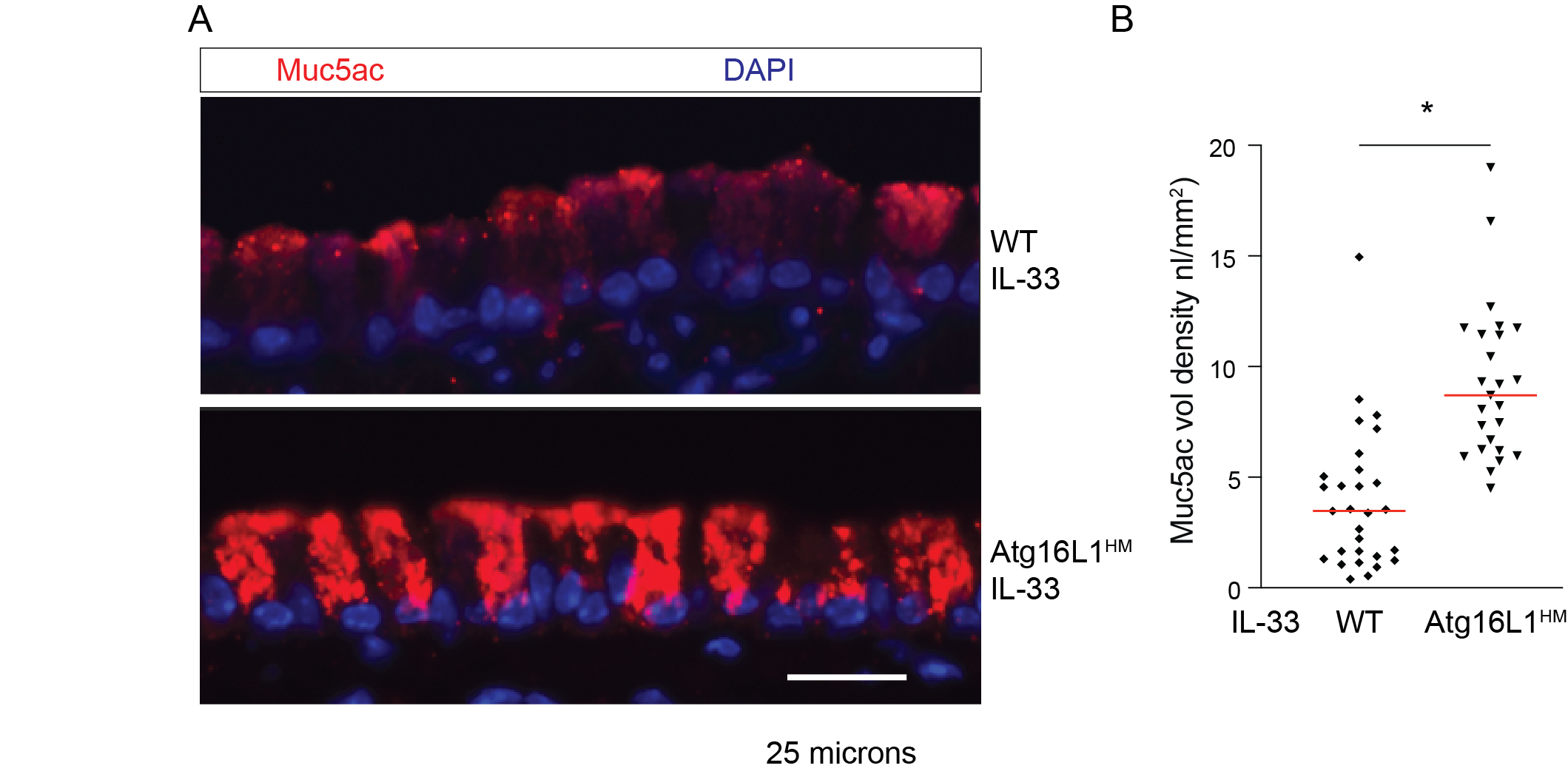


**Figure S2**: **Atg16L1HM mice have increased intracellular Muc5ac by immunofluorescence after IL-33 -mediated mucous metaplasia. (A)** Representative Muc5ac immunostaining for WT and Atg16L1 airways using rabbit polyclonal antibody UNC294. (**B**) Graphs show scatter plots with median bar. N=3 mice per group (8-10 images per sample. Significant difference by unpaired T-test with * for mouse genotype difference. Scale bar=25 microns.


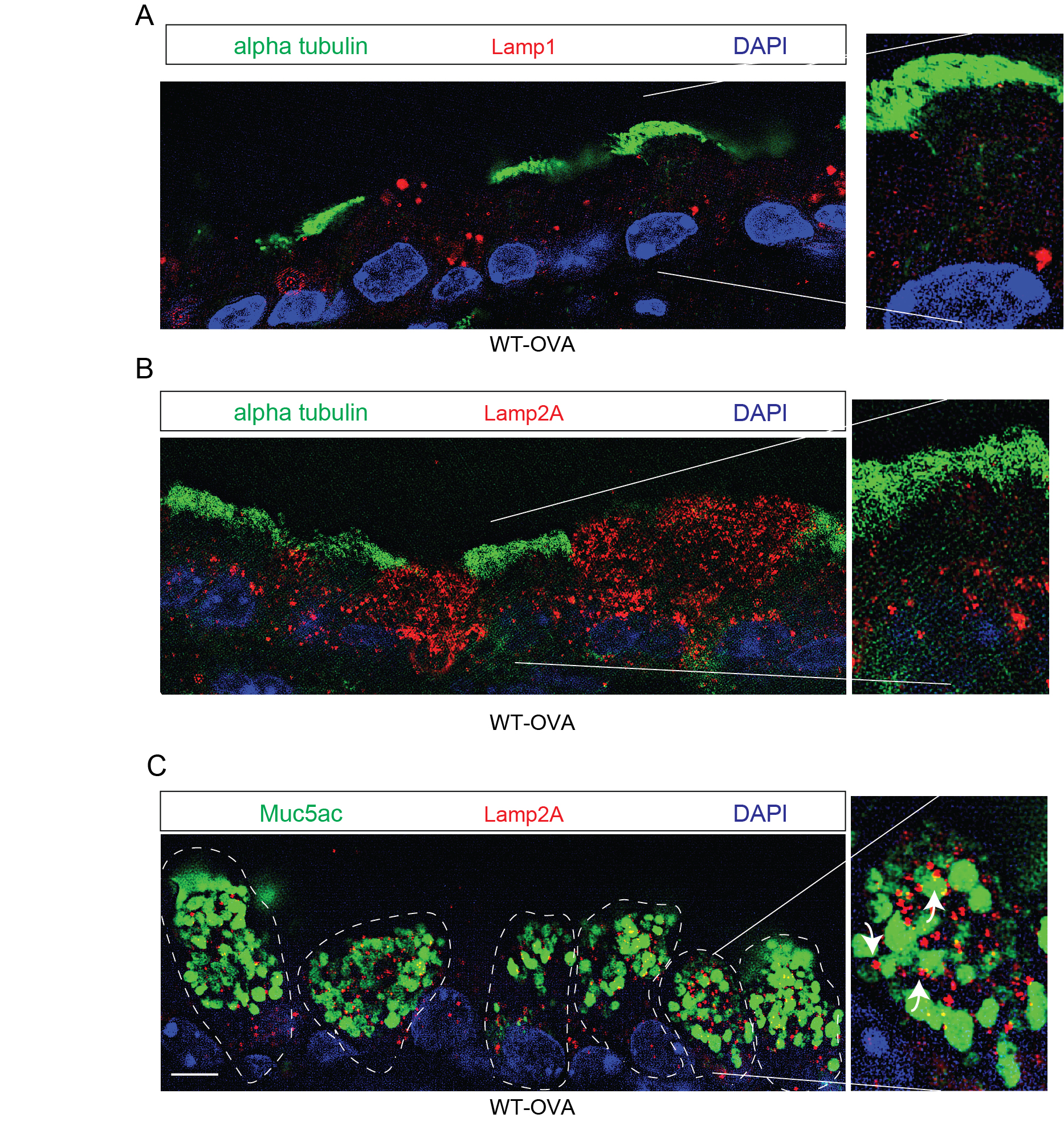
**Figure S3**: **Lysosomal membrane LAMP proteins concentrate in the cytoplasm surrounding mucin granules of secretory cells.** (**A**) Lamp1 (red), acetylated (AC) alpha tubulin (green), immunostaining in OVA challenged mice. Representative Lamp2A (red), acetylated alpha tubulin (green) (**B**) or Muc5ac by lectin UEA-1 (green) (**C**) staining in OVA challenged mouse airways. challenged mice. Scale bar =5 microns. DAPI for nuclear counter staining. Arrows in inset point to Lamp2A adjacent to or overlapping with Muc5ac granules.

**
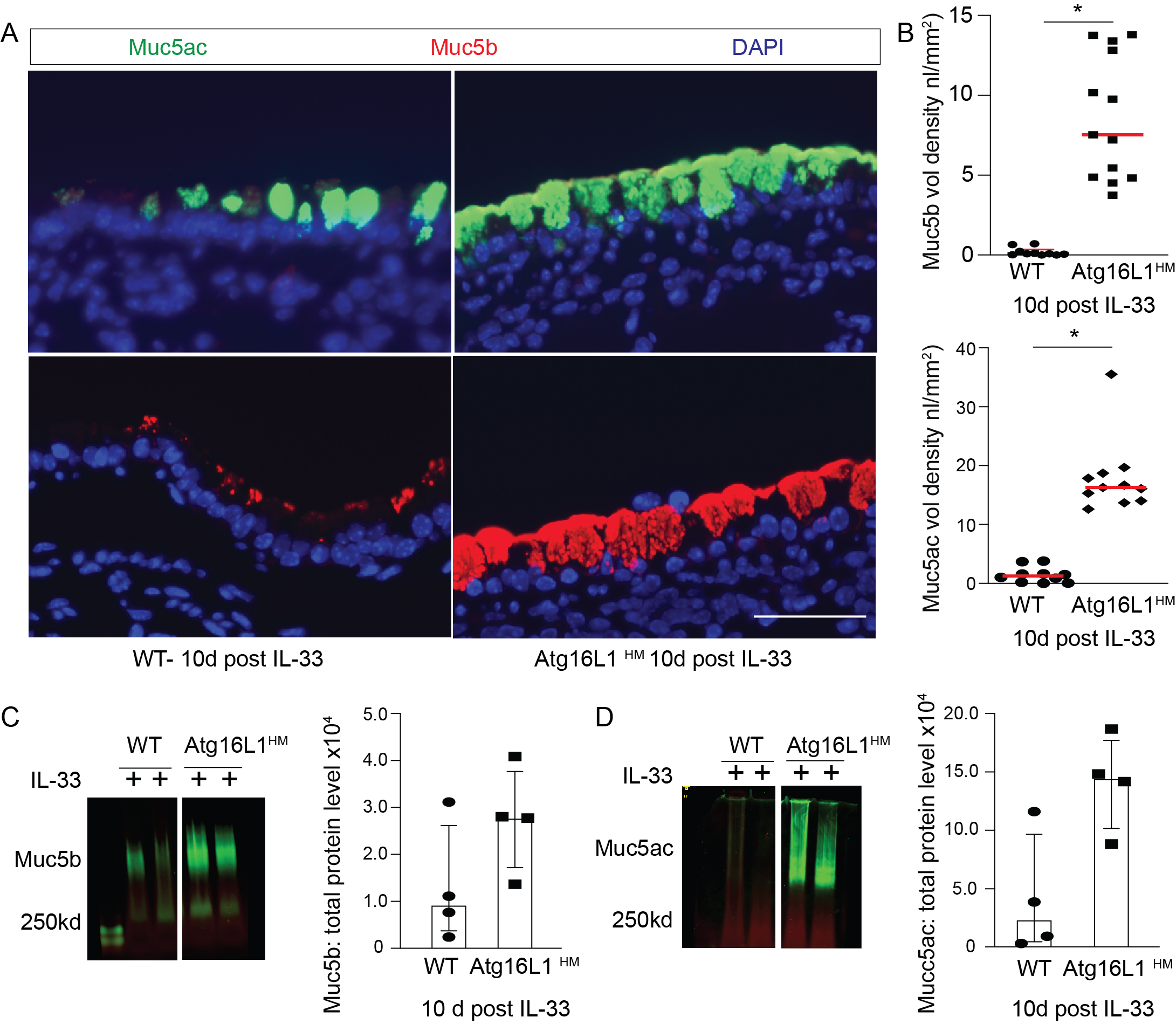
 Figure S4: (A,B**) **Atg16L1 deficient mice have slower resolution following IL-33 induced airway mucous metaplasia.** Representative immunostaining and quantification for Muc5ac using lectin UEA-1 and Muc5b 10 days following last of 3 challenges of intra-nasal IL-33. Scale bar equals 20 microns. **C,D**) Representative images of immunoblots of Muc5b and Muc5ac by lectin UEA-1 in WT and Atg16L1^HM^ mouse airways at 10 days following last IL-33 challenge with corresponding quantification and band density values normalized to total protein levels (n=4 mice per group). Graphs show scatter plots with median bar and interquartile range for mucin blots and scatter blot for mucin immunostaining data with median line. Significant difference by unpaired T-test for immunostaining data in part **B**.

**
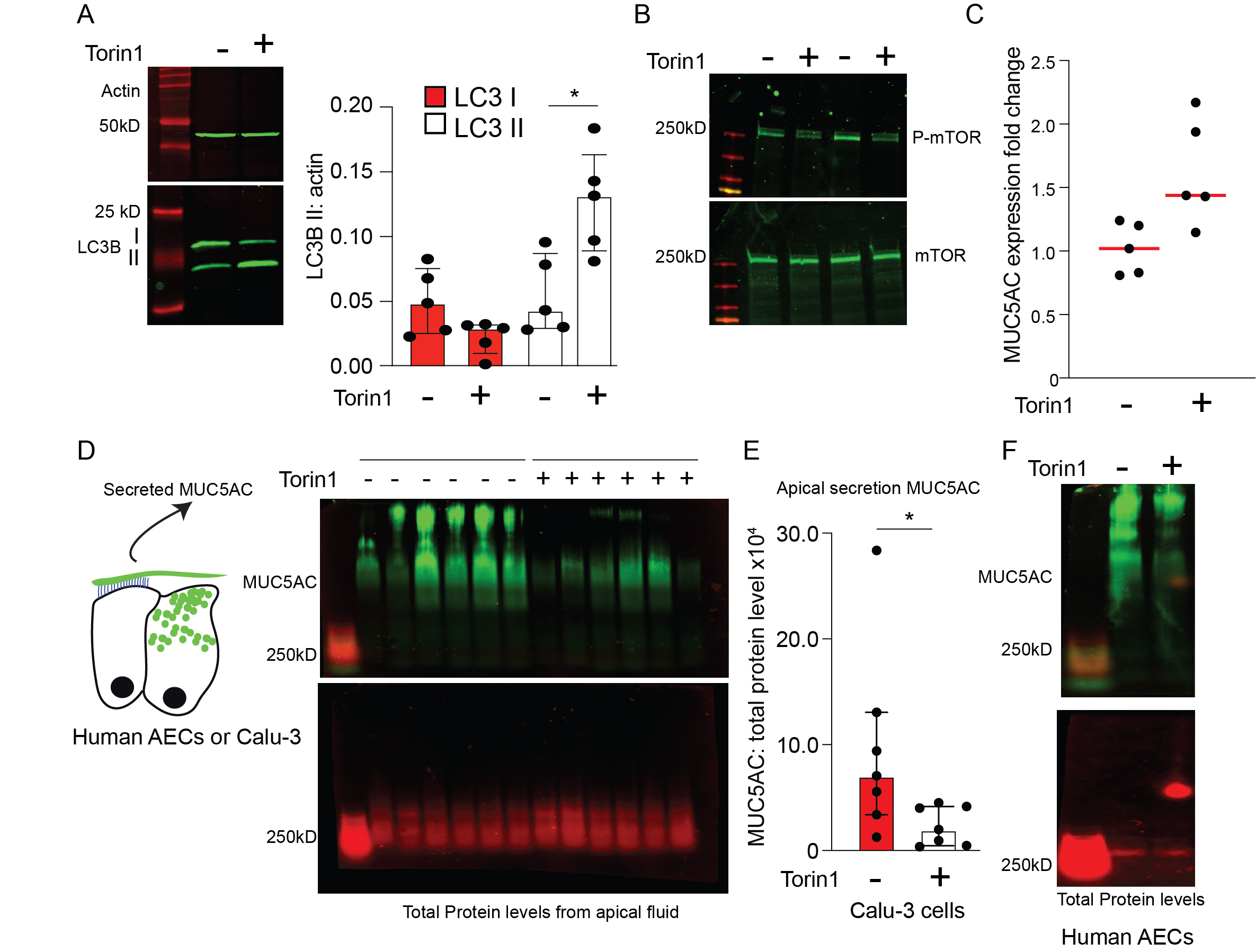
Figure S5**: **Torin1 activates autophagy by mTOR inhibition, however, does not alter MUC5AC expression or increase apical secretion.** **A**) Representative LC3B immunoblot from Calu-3 cells under ALI conditions given torin1 (10μM) for 18hr. LC3B I and II levels were each normalized to total protein. N=5 inserts per group. **B)** Representative immunoblot for mTOR and phosphorylated mTOR (S2448) with and without torin1 in duplicate conditions. **C**) MUC5AC expression from Calu-3 ±torin1 (10μM) 18hr. Expression normalized by OAZ1 and reported as fold change over untreated (N=5 per group). (**D**) Calu-3 cells under ALI conditions were washed with PBS to remove residual mucous layer, then treated with torin1 10μM or vehicle for 18hr. Immunoblot of secreted MUC5AC levels from apical surface with corresponding quantification normalized to total protein (**E**). (**F**) Representative immunoblot of secreted MUC5AC from human AECs treated with torin 10μM or vehicle for 18hr as shown in part (**D,E**). Graphs show scatter plots with median bar and interquartile range for LC3 and MUC5AC western blots and scatter blots for MUC5AC expression data with median line. Mann Whitney test was used to test significance of LC3 western blot data (**A**) and MUC5AC expression (**C)** and MUC5AC secreted protein data (**E**).

**
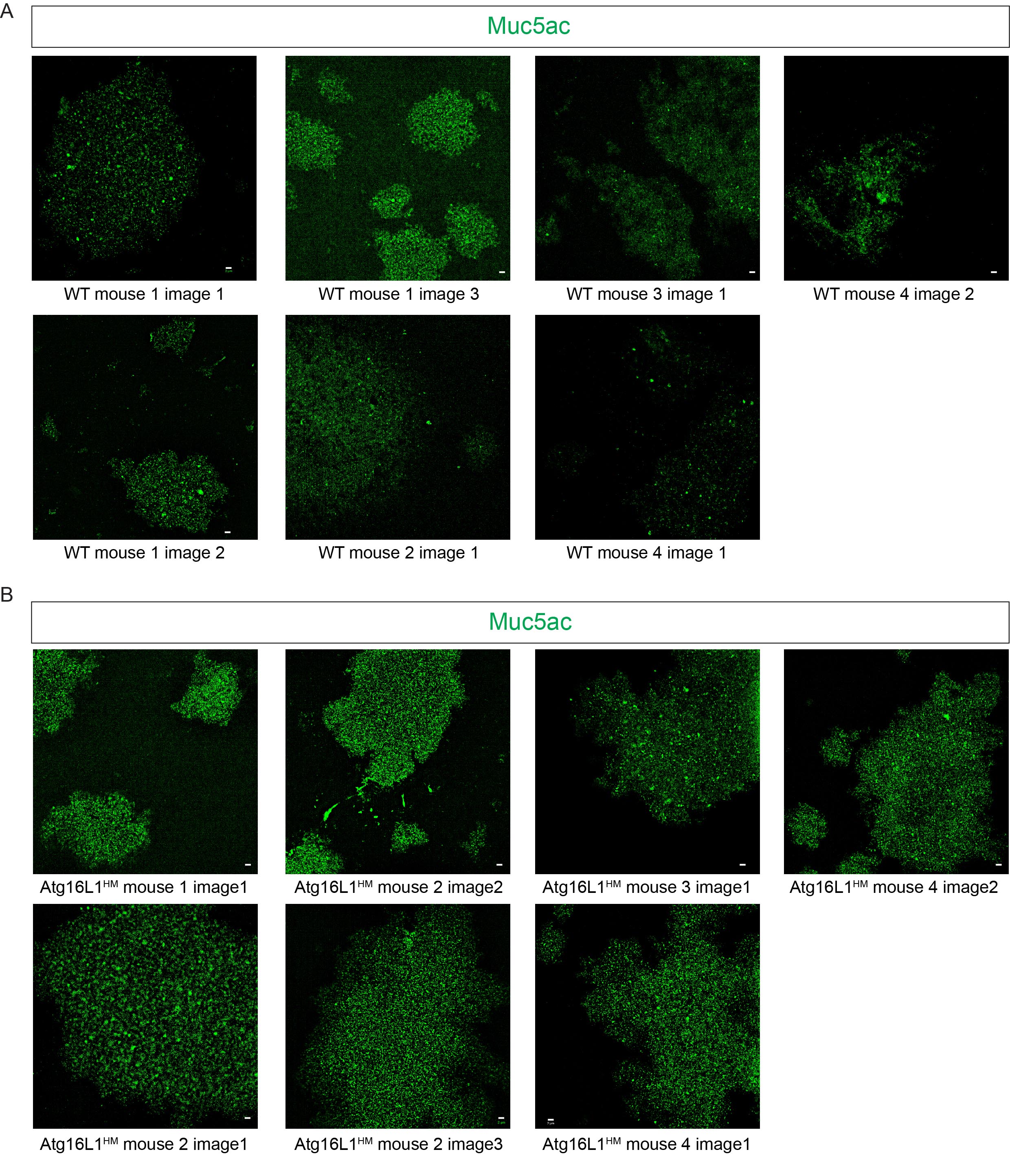
Figure S6: Lysosome enriched preparations from Atg16L1 deficient mice have increased number of mucin granules compared to WT mice at day 3 of resolution from OVA-mediated mucous metaplasia. A)** Seven images from lysosome enriched preparations 4 different WT mice. **B**) Seven images from lysosome enriched preparations 4 different Atg16L1^HM^ mice. Scale bar =2 microns.

**Title: Autophagy of mucin granules contributes to resolution of airway mucous metaplasia.**

**Authors**: Sweeter, JM^1^, Kudrna K^1^, Hunt, K^1^, Thomes, P^1^, Dickey, BF^2^, Brody SL^3^, Dickinson, JD*^1^

Uncropped Gel images:

Figure 1:


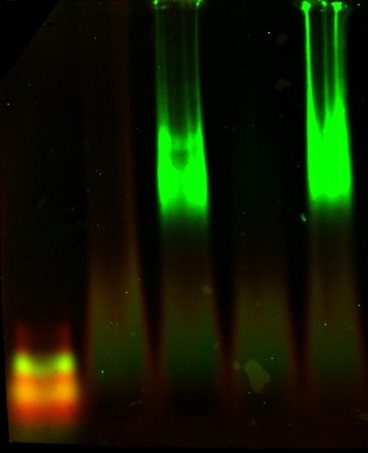
Part D Part E


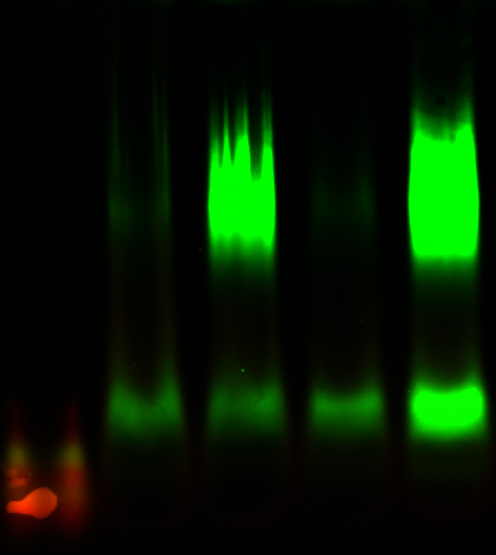


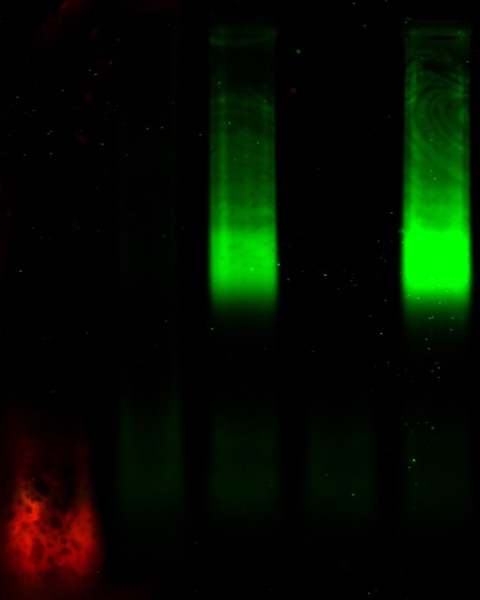

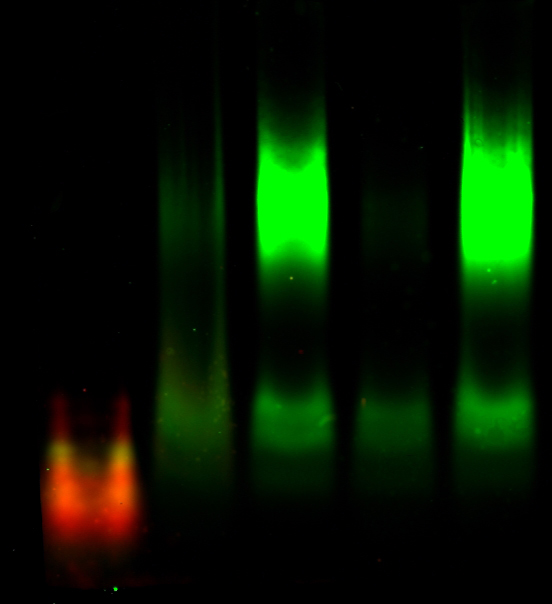
Figure 2:

Figure 3:

Part A


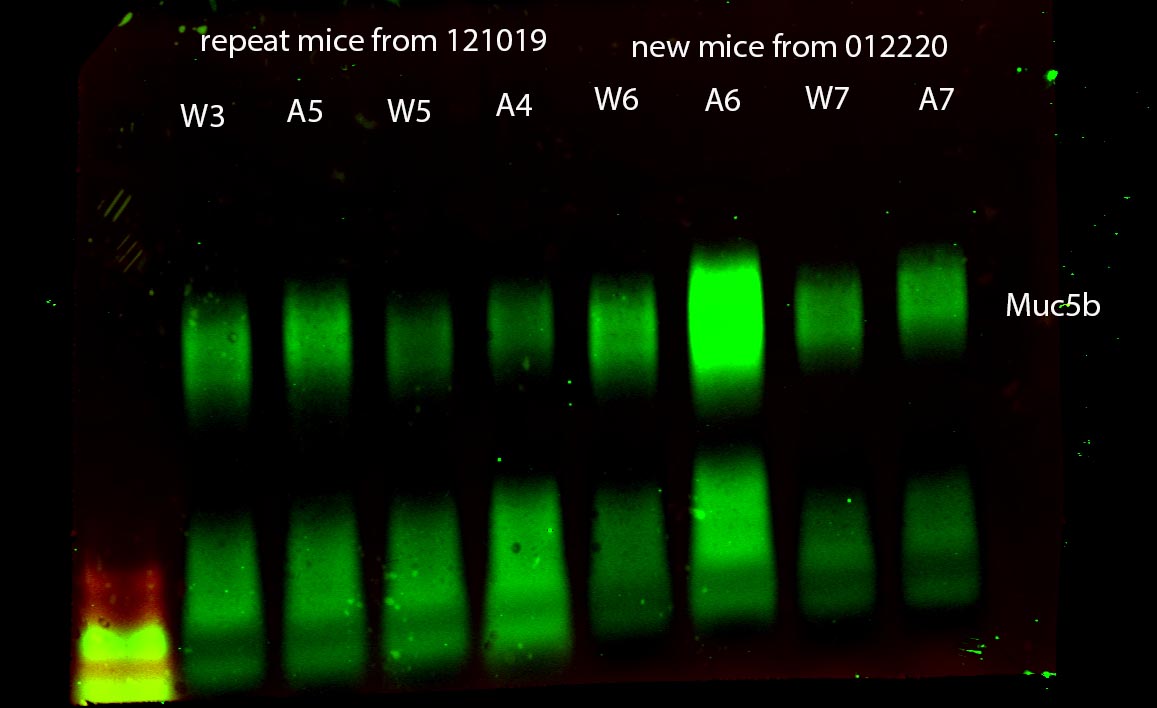


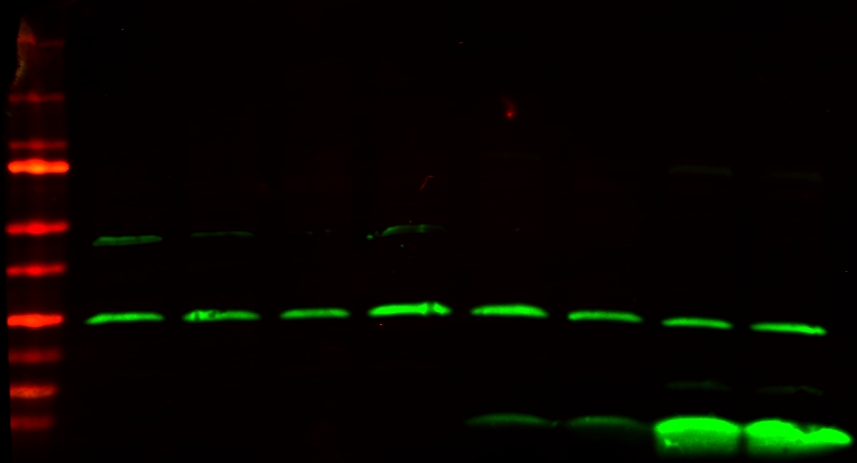
Part C

Figure 5:


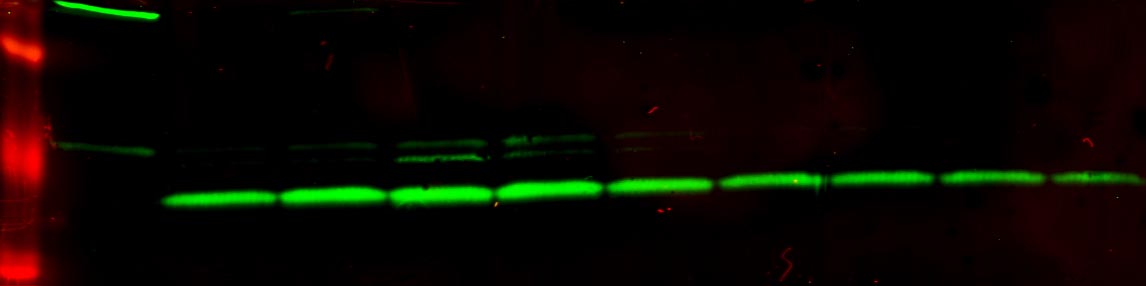
Part A


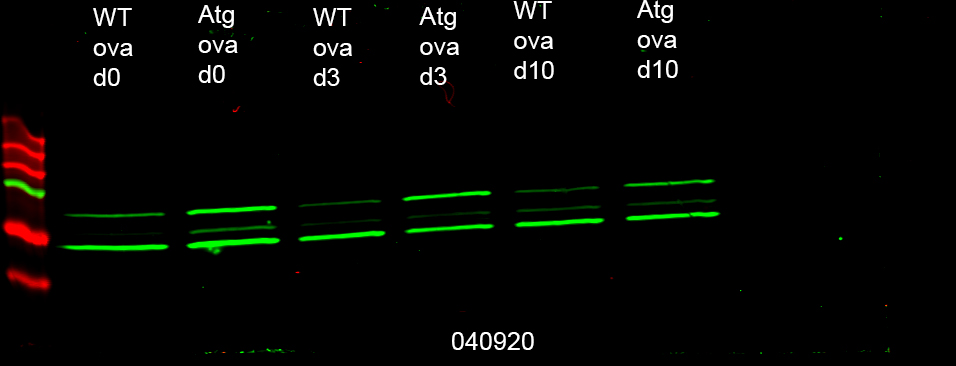
 Part D upper gel

Part D lower gel


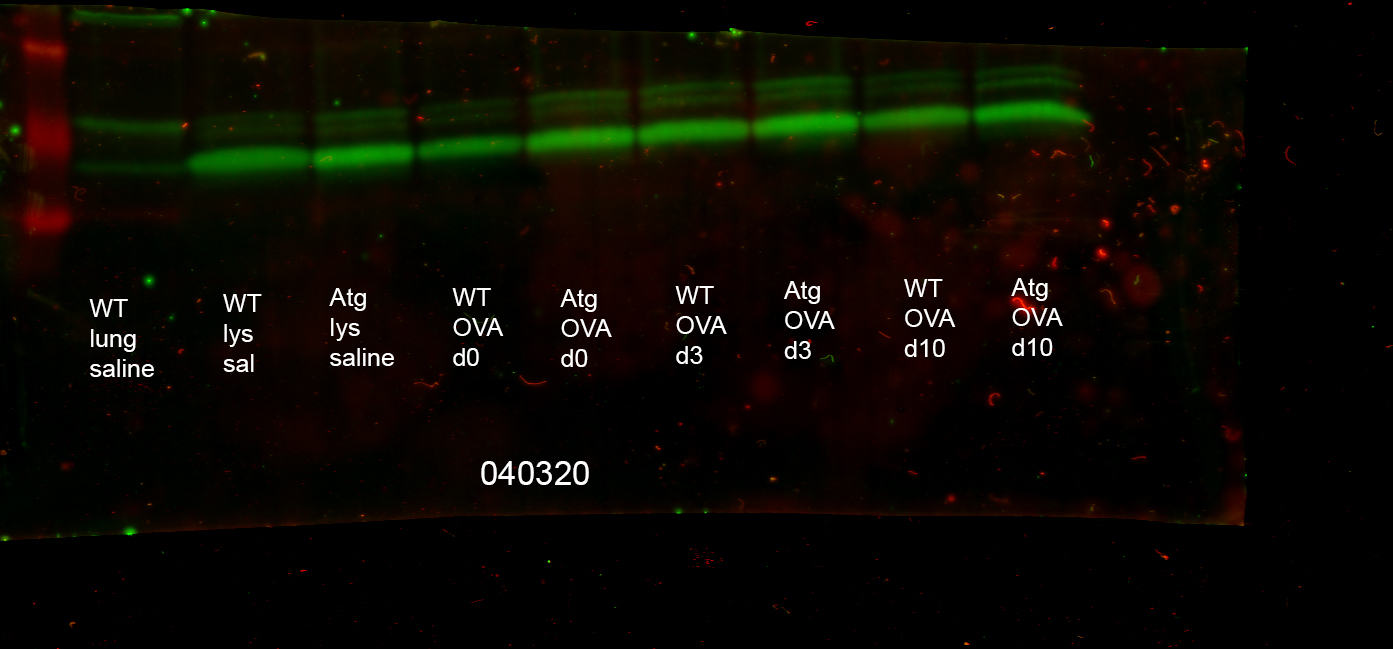


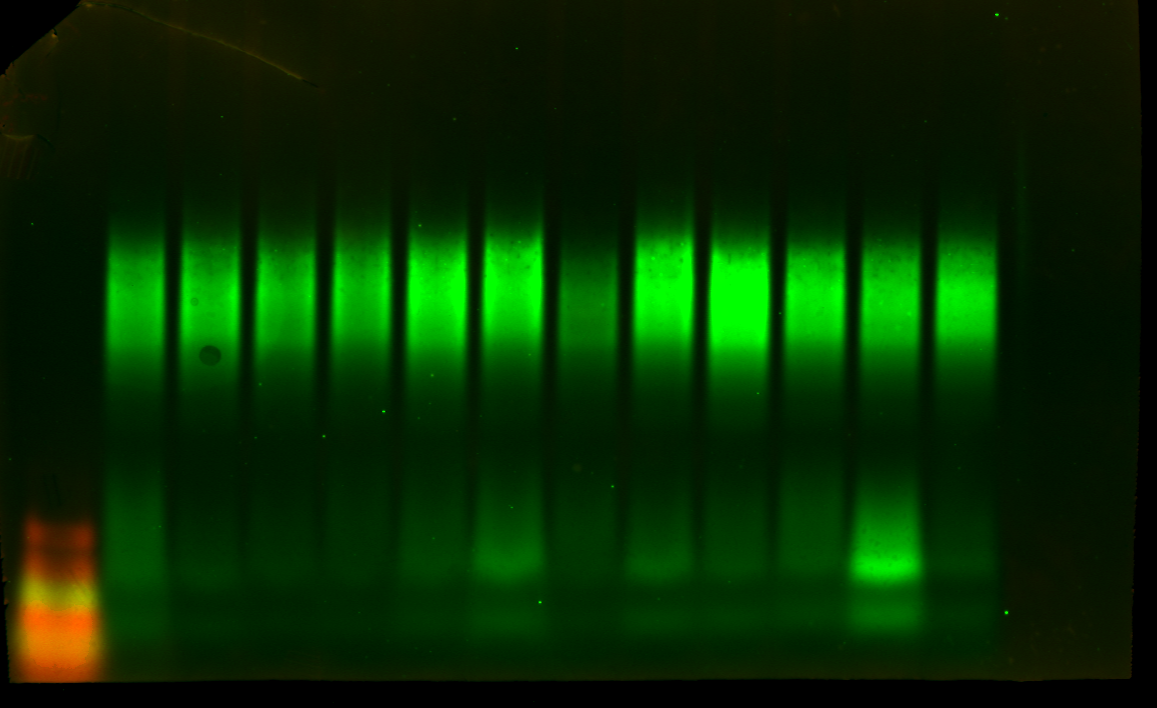
Part G

Part H


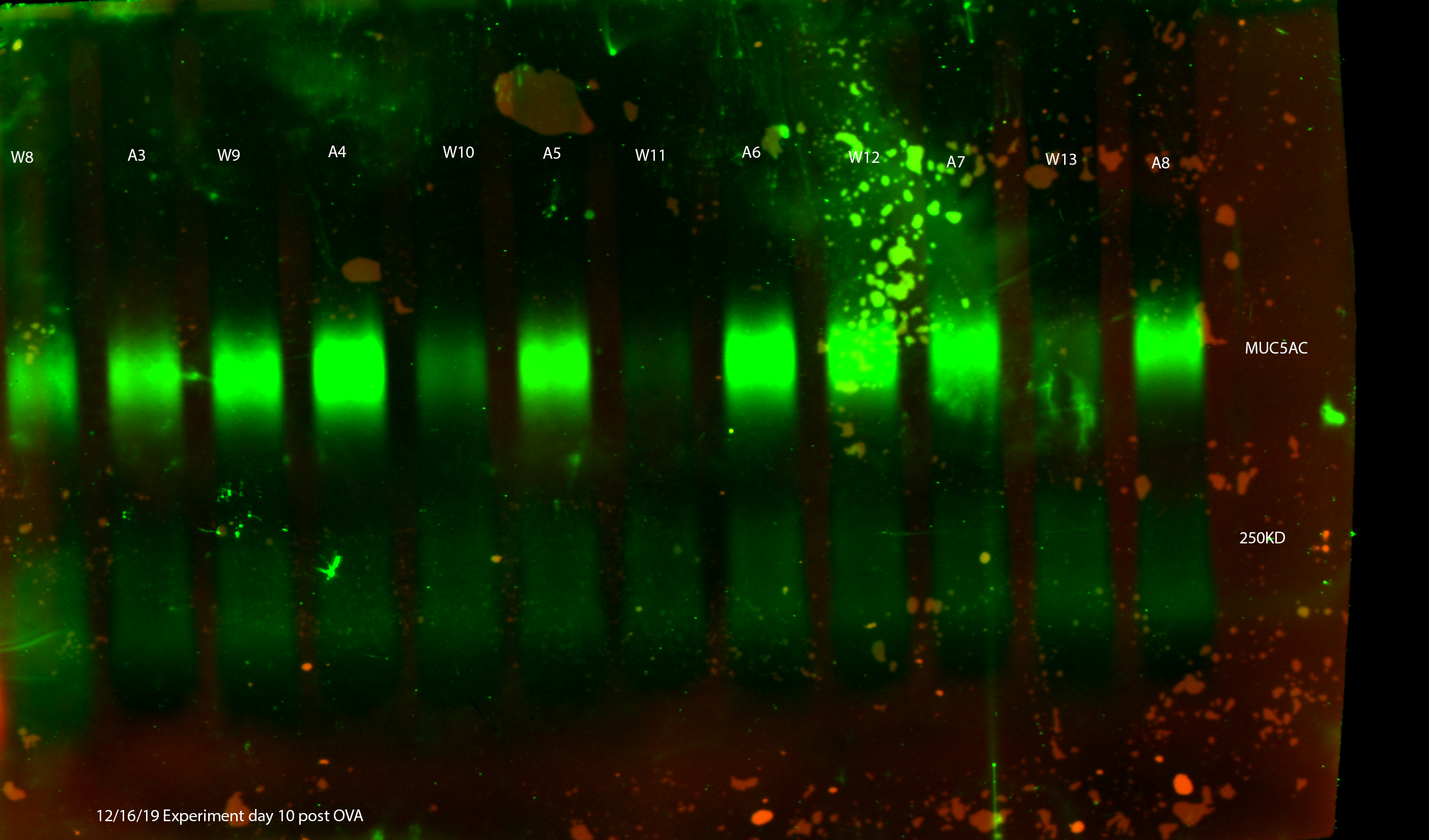


Figure 6


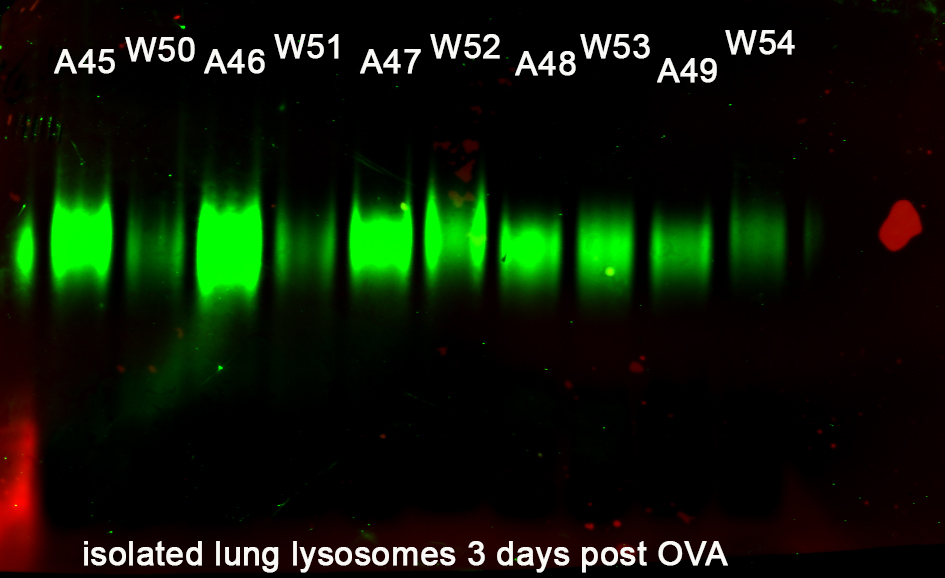
Part B


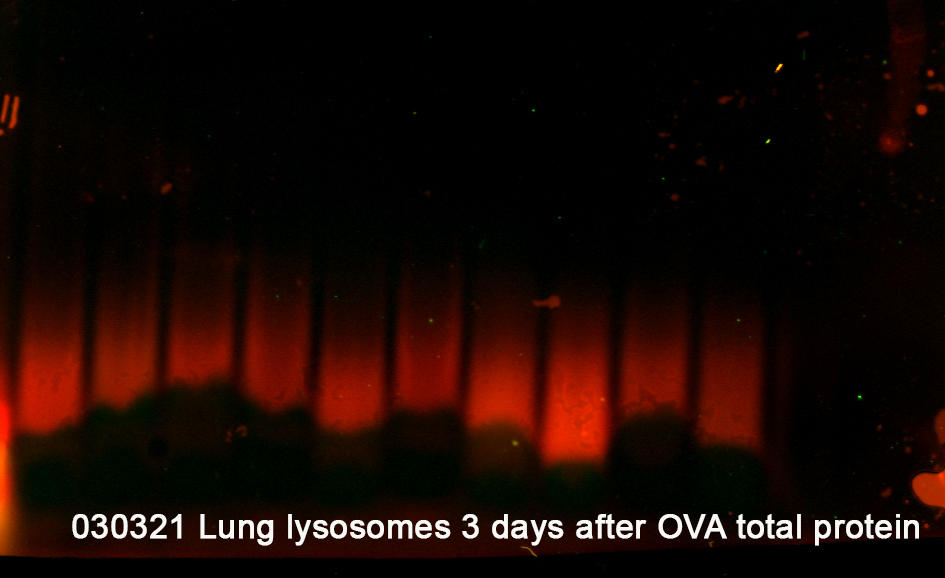


Figure 7:


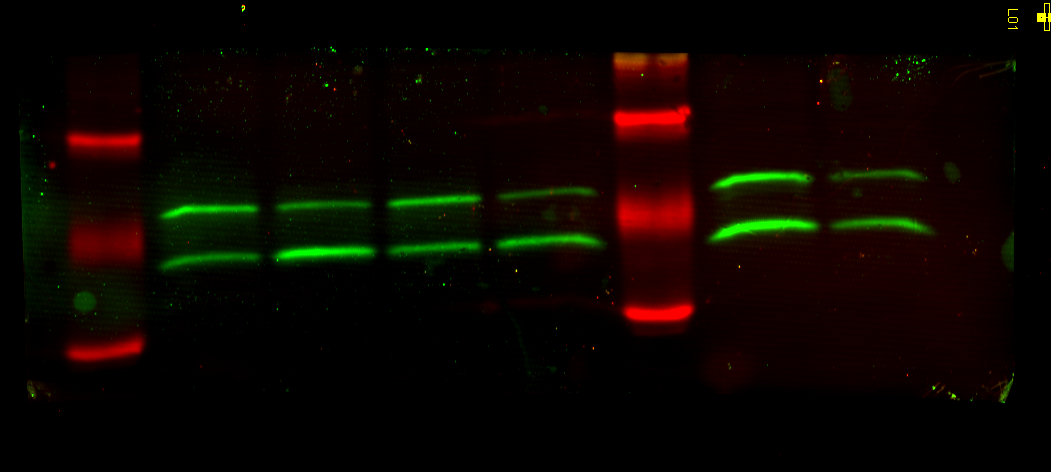
Part B

Part C


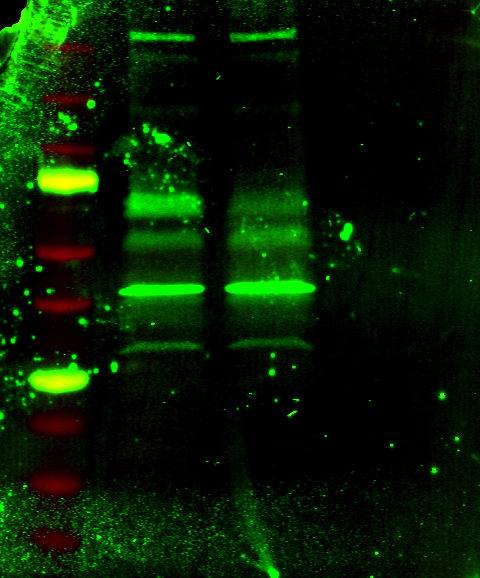


Figure 8

Part A


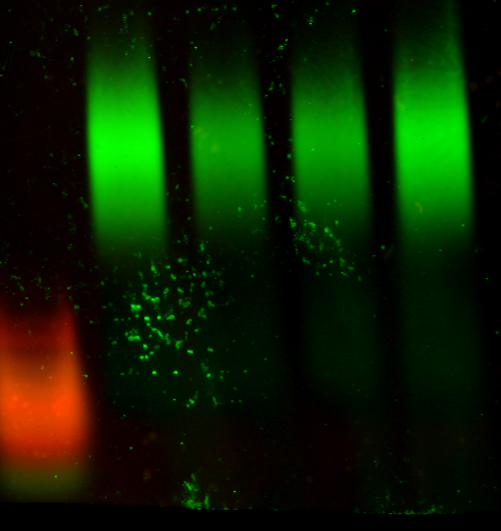


Figure 9:

Part A


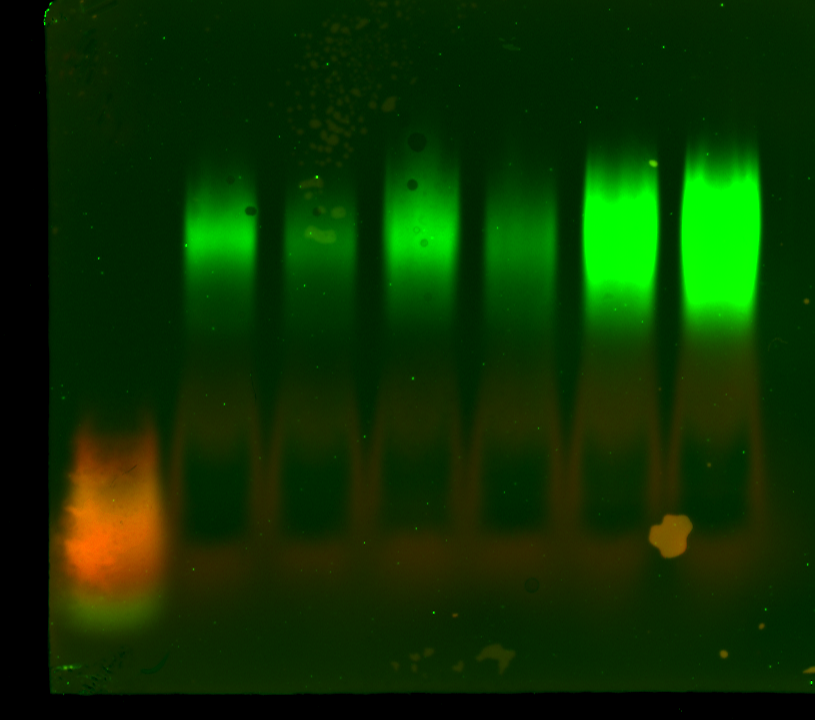


Part C


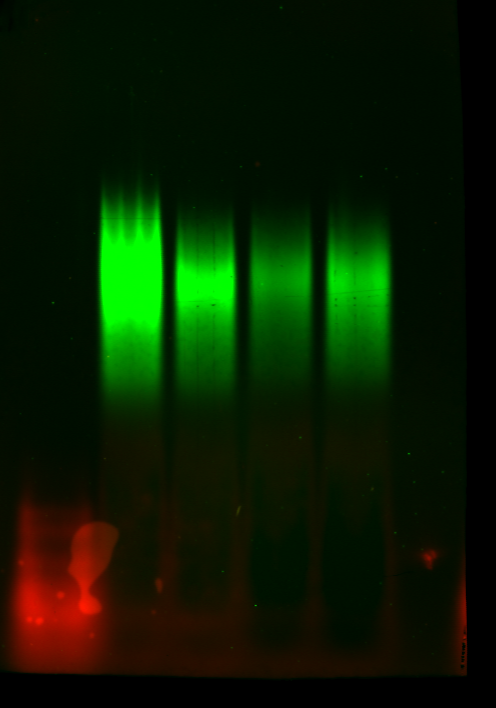


Supplemental Figure1

Part A


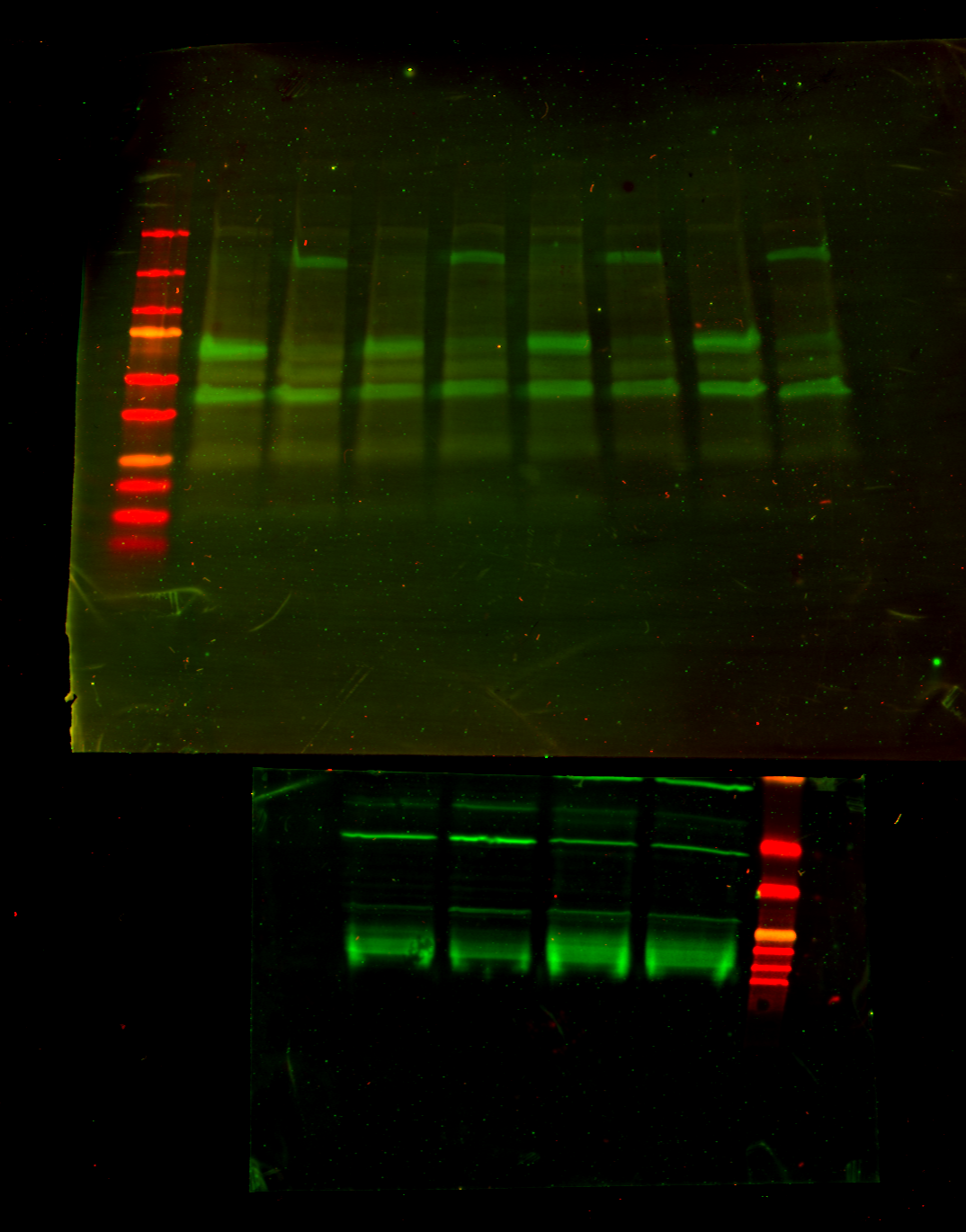


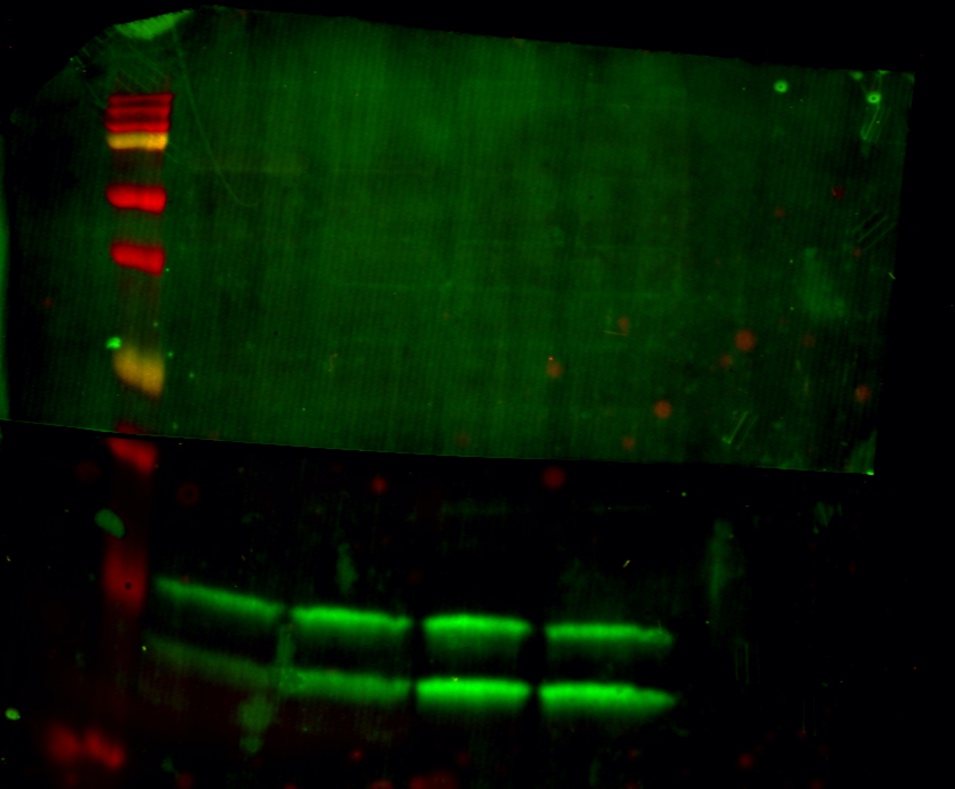
Part B


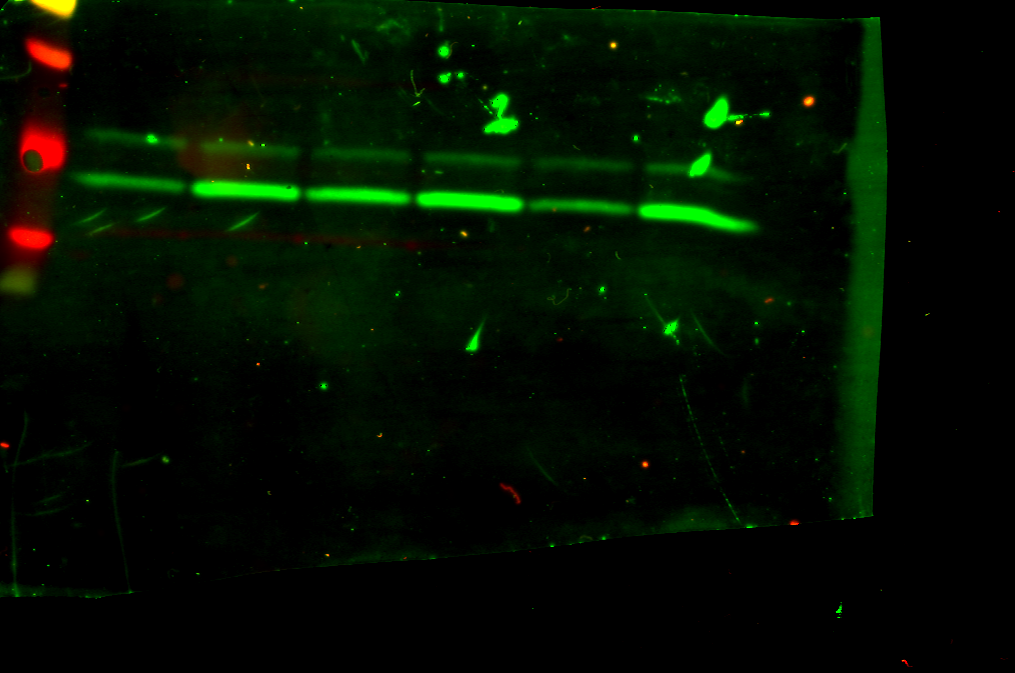
Part C (lower gel) Part C ( upper gel)


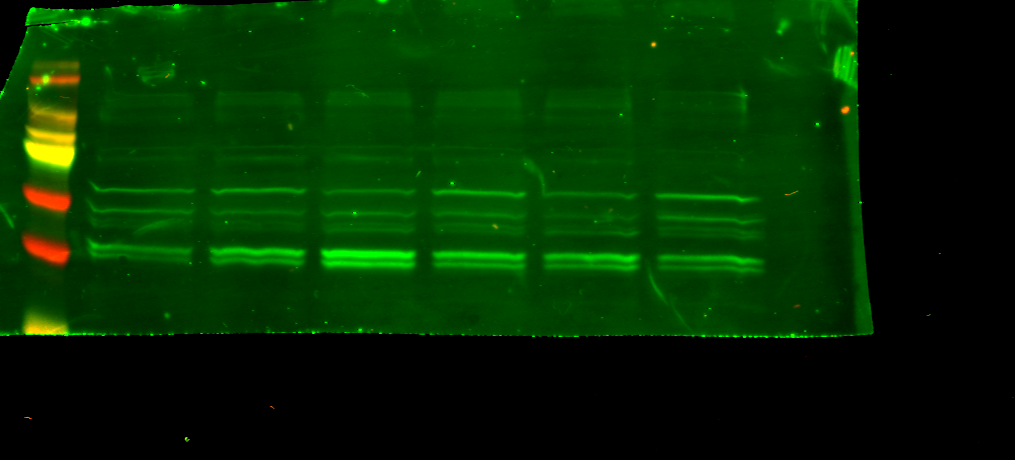


Supplemental Figure4

Part C


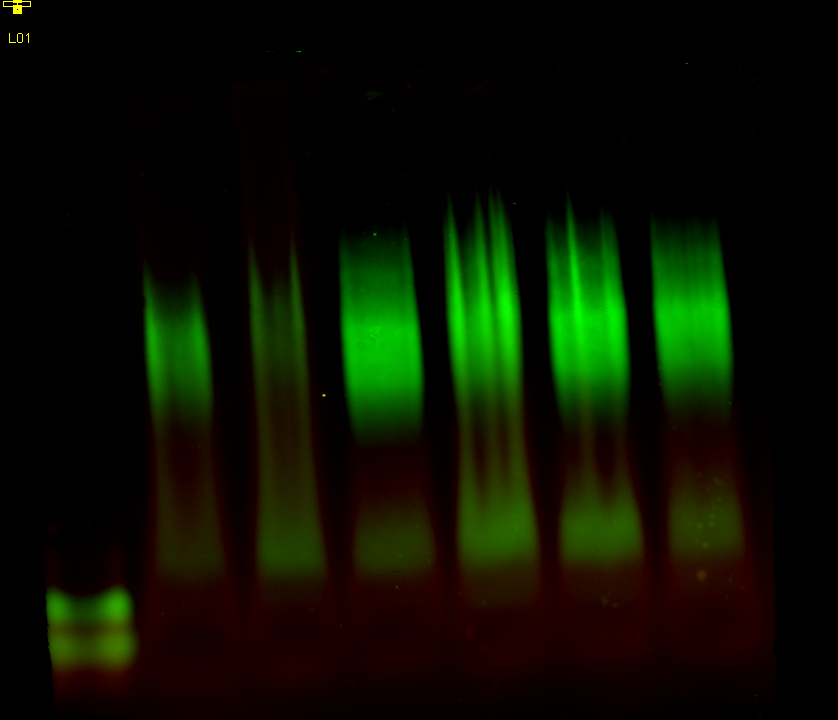


Part D


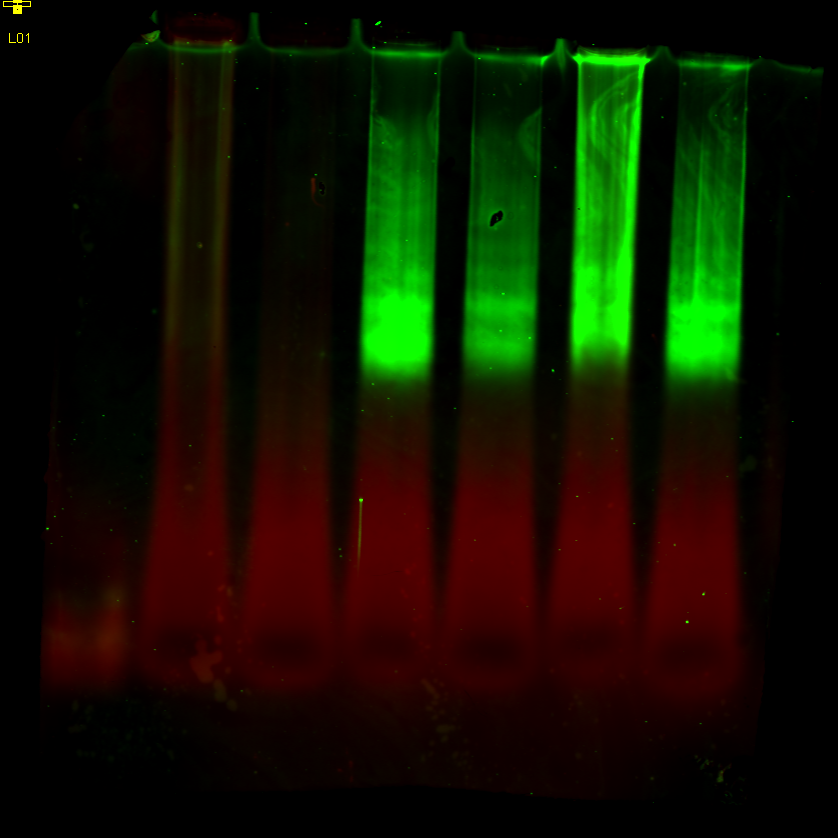


Supplemental Figure5

Part A Part B upper panel


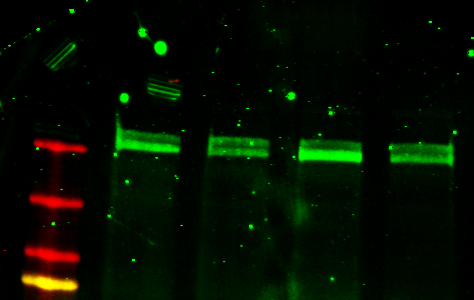

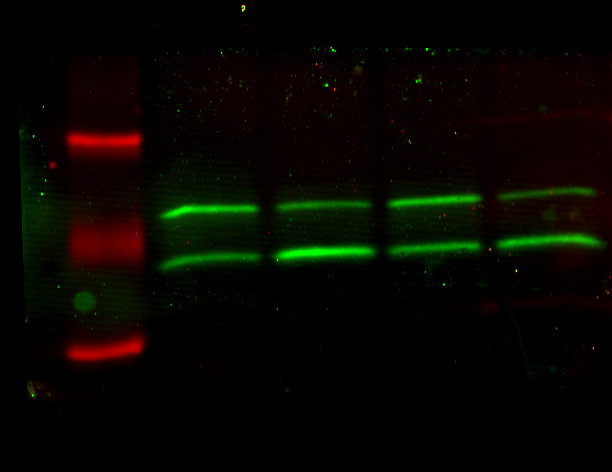


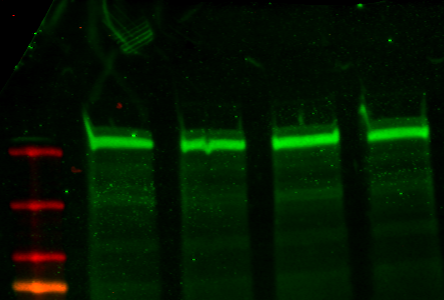
Part B lower panel


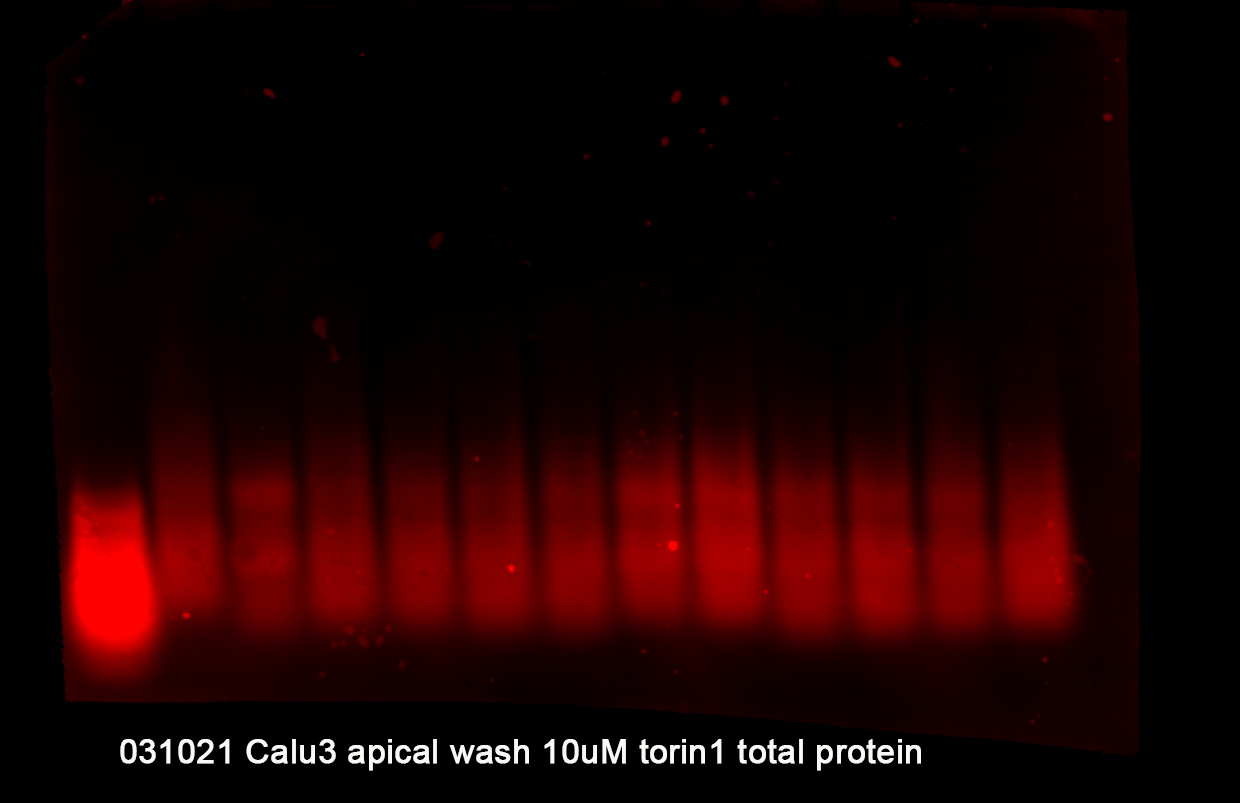

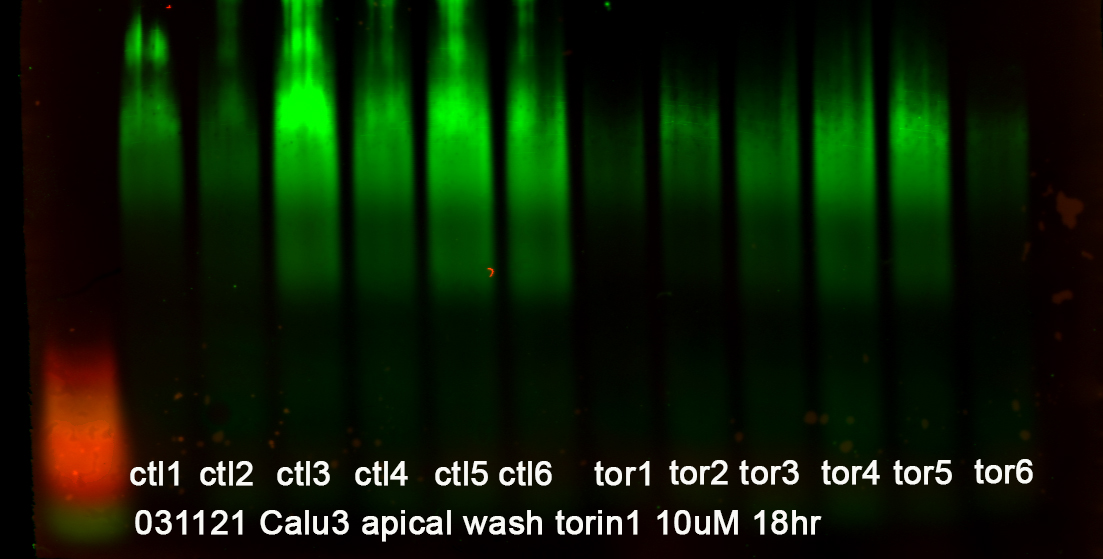
Part D


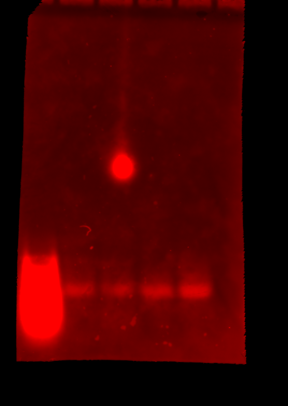

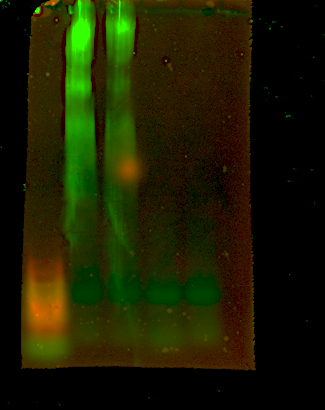
Part E
